# Supplementary material for: Sectoral sensitivity of the Kuwait stock market to a dual shock
Source: PLoS One. 2025 Sep 24;20(9):e0331384. doi: 10.1371/journal.pone.0331384 (PMC12459840; doi:10.1371/journal.pone.0331384)
Supplement: S1 Table — (DOCX) [file pone.0331384.s001.docx]

| **Correlation Prices** | | | | | | | | | | | | | |
| --- | --- | --- | --- | --- | --- | --- | --- | --- | --- | --- | --- | --- | --- |
|  | **All share** | **Banks** | **REAL ESTATE** | **Financial Services** | **Telecom** | **Consumer Services** | **OIL & Gas** | **Healthcare** | **Insurance** | **Basic Materials** | **Consumer Goods** | **Industrials** | **Technology** |
| **WTI** | 0.64975 | 0.53671 | 0.59365 | 0.61434 | 0.32937 | 0.30569 | 0.59713 | 0.33651 | 0.60009 | 0.73750 | -0.33326 | 0.82140 | -0.21051 |
| **Brent** | 0.62971 | 0.53035 | 0.48469 | 0.54028 | 0.28730 | 0.24745 | 0.58607 | 0.41921 | 0.52271 | 0.80923 | -0.40316 | 0.82573 | -0.27062 |
| **OPEC** | 0.66930 | 0.57755 | 0.47321 | 0.53347 | 0.33738 | 0.20974 | 0.62607 | 0.44807 | 0.52182 | 0.83074 | -0.44524 | 0.83700 | -0.32407 |
| **DUBAI** | 0.65755 | 0.56394 | 0.46362 | 0.52885 | 0.32386 | 0.20127 | 0.62212 | 0.46270 | 0.52141 | 0.83771 | -0.44401 | 0.84199 | -0.32187 |

S1Table. Correlation Findings for Prices

***Note:*** *This table reports the correlation matrix of daily prices for Kuwait stock market sectors and oil benchmarks. The research sample under consideration spans from December 31 2015 to February 23 2022. All variables are positively correlated with different percentages, except for Consumer Goods and Technology, which recorded no correlation. Source: Data Stream (2023)*

|  | | | | | | | | | | | | | |
| --- | --- | --- | --- | --- | --- | --- | --- | --- | --- | --- | --- | --- | --- |
|  |  |  |  |  |  |  |  |  |  |  |  |  |  |
|  |  |  |  |  |  |  |  |  |  |  |  |  |  |
|  |  |  |  |  |  |  |  |  |  |  |  |  |  |
|  |  |  |  |  |  |  |  |  |  |  |  |  |  |
|  |  |  |  |  |  |  |  |  |  |  |  |  |  |


$Z_{t}=A_{1}Z_{t-1}+A_{2}Z_{t-2}+\cdots+A_{k}Z_{t-k}+\mu_{t}$ $\Delta Z_{t}=\Gamma_{1}\Delta Z_{t-1}+\Gamma_{2}\Delta Z_{t-2}+\cdots+\Gamma_{k-1}\Delta Z_{t-k-1}+\Pi Z_{t-1}+\mu_{t}$ $\Gamma_{i}=\left( I-A_{1}-A_{2}-\cdots-A_{k} \right)(i=1,2,\ldots,k-1)$ $\Pi=-\left( I-A_{1}-A_{2}-\cdots-A_{k} \right)$ $y_{t}=\alpha_{1}+\sum_{i=1}^{n} \beta_{i}x_{t-i}+\sum_{j=1}^{m} \gamma_{j}y_{t-j}+\varepsilon_{1t}$ $x_{t}=\alpha_{2}+\sum_{i=1}^{n} \theta_{i}x_{t-i}+\sum_{j=1}^{m} \delta_{j}y_{t-j}+\varepsilon_{2t}$ $y_{t}$ $x_{t}$ $y_{t}$ $Y_{t}$ $X_{t}$ $\Theta\left( L \right)\left( \begin{aligned} &Y_{t} \\ &X_{t} \end{aligned} \right)=\left( \begin{aligned} &\Theta_{11}(L) &&\Theta_{12}(L) \\ &\Theta_{21}(L) &&\Theta_{22}(L) \end{aligned} \right)\left( \begin{aligned} &Y_{t} \\ &X_{t} \end{aligned} \right)=\varepsilon_{t}$ $\Theta(L)=I-\Theta_{1}L-\cdots-\Theta_{p}L_{p}$ $\Theta_{1},\ldots,\Theta_{p}$ $L^{k}X_{t}=X_{t-k} and L^{k}Y_{t}=Y_{t-k}$ $\varepsilon_{t}$ $\left( \varepsilon_{t}\varepsilon_{t}^{t} \right)$ $\left( \begin{aligned} &Y_{t} \\ &X_{t} \end{aligned} \right)=\psi(L)\eta_{t}=\left( \begin{aligned} &\psi_{11}(L) &&\psi_{12}(L) \\ &\psi_{21}(L) &&\psi_{22}(L) \end{aligned} \right)\left( \begin{aligned} &\eta_{1t} \\ &\eta_{2t} \end{aligned} \right)$ $M_{X\Rightarrow\gamma}(Y)=log\left[ 1+\frac{\left| \psi_{12}\left( e^{-i\gamma} \right) \right|^{2}}{\left| \psi_{11}\left( e^{-i\gamma} \right) \right|^{2}} \right]$ $\left| \Theta_{12}\left( e^{-i\gamma} \right) \right|=\left| \sum_{k=1}^{p} \Theta_{k,12}cos(k\gamma) \right|-i\sum_{k=1}^{p} \Theta_{k,12}sin(k\gamma)\mid=0$ $\Theta_{k,12}$ $\Theta_{k,}$ $\sum_{k=1}^{p} \Theta_{k,12}cos(k\gamma)\mid=0 and \sum_{k=1}^{p} \Theta_{k,12}sin(k\gamma)=0$
