# Supplementary material for: Sectoral sensitivity of the Kuwait stock market to a dual shock
Source: PLoS One. 2025 Sep 24;20(9):e0331384. doi: 10.1371/journal.pone.0331384 (PMC12459840; doi:10.1371/journal.pone.0331384)
Supplement: S2 Table — (DOCX) [file pone.0331384.s002.docx]

| **Correlation Returns** | | | | | | | | | | | | | |
| --- | --- | --- | --- | --- | --- | --- | --- | --- | --- | --- | --- | --- | --- |
|  | **All share** | **Banks** | **REAL ESTATE** | **Financial Services** | **Telecom** | **Consumer Services** | **OIL & Gas** | **Healthcare** | **Insurance** | **Basic Materials** | **Consumer Goods** | **Industrials** | **Technology** |
| **WTI** | 0.16244 | 0.14622 | 0.06858 | 0.10860 | 0.13617 | 0.09005 | 0.04706 | 0.03446 | 0.00012 | 0.08043 | 0.05113 | 0.16696 | -0.00899 |
| **Brent** | 0.19629 | 0.17720 | 0.12741 | 0.16761 | 0.10867 | 0.10427 | 0.01811 | 0.02802 | -0.00191 | 0.10136 | 0.06307 | 0.22001 | -0.01597 |
| **OPEC** | 0.21490 | 0.18603 | 0.15816 | 0.17261 | 0.16526 | 0.09815 | 0.02775 | 0.01627 | 0.01440 | 0.12290 | 0.10624 | 0.21199 | 0.00162 |
| **DUBAI** | 0.20694 | 0.18812 | 0.15225 | 0.16949 | 0.11541 | 0.09816 | 0.02327 | 0.02064 | -0.00194 | 0.10554 | 0.07640 | 0.22706 | -0.00121 |

S2Table. Correlation Findings for Returns

***Note:*** *This table reports the correlation matrix of daily Returns for Kuwait stock market sectors and oil benchmarks. The research sample under consideration spans from December 31 2015 to February 23 2022. All variables are positively correlated with different percentages, except for Insurance and Technology, which showed no correlation. Source: Data Stream (2023)*
