# Supplementary material for: Sectoral sensitivity of the Kuwait stock market to a dual shock
Source: PLoS One. 2025 Sep 24;20(9):e0331384. doi: 10.1371/journal.pone.0331384 (PMC12459840; doi:10.1371/journal.pone.0331384)
Supplement: S1 File — (DOCX) [file pone.0331384.s003.docx]

**S1 Methodology Description**

**3.2.1 Stationarity Testing**

Time series are stationary when their statistical properties, mean, variance, and autocorrelation are stable. Before modelling cointegration and causality, pretesting is required to ensure a stationary relationship among the variables. The series stationarity properties were examined by implementing three well-known tests: The Augmented Dickey-Fuller (ADF) test (1979), Phillips-Perron (PP) test (1988), and Kwiatkowski-Phillips-Schmidt-Shin (KPSS) (Kwiatkowski et al. 1992). We do not assume the series behaviour to be a pure random walk in financial markets, as the drift captures the variation level. Besides, in the literature, Al-Jafari (2011) examined the random walk theory on the Kuwait stock market, and the study results confirm that the Kuwait stock market is not a pure random walk (Alshogeathri, 2011; Al-Nassar,2021; Al-Refai, Zaitun & Eissa 2022; Verma et al., 2023). Appendix 2 Tables 1 and 2 present the outcomes for the unit root test using the ADF, PP, and KPSS tests. The tests show that in the price series for Kuwait’s sectors, oil benchmarks are non-stationary in levels but stationary at a 1 percent level in returns. Moreover, the p-values results are shown in parentheses; there is no p-value for KPSS; therefore, the 1 percent significance level was considered for the test at a value of 0.739000. The main indices for the sectors and major oil Benchmarks are presented, and the results for the three stationarity tests with p-values in brackets and the number of required lags to estimate the tests are reported in Appendix 2 Tables 1 and 2.

- - 1. **Cointegration**

The selected research methodology offers insights into the relationship characterising Kuwait’s stock market sectors and oil benchmarks. The Johansen Cointegration test (Johansen, 1988) is applied in combination with Engle and Granger Cointegration (1987) approach, therefore providing an opportunity to cross-check results in terms of robustness and validity of research findings. The Johansen (1988) approach extends the single equation error correction model to a multivariate one. For instance, if we suggest that there are three endogenous variables yt, xt and wt, and the matrix notion is Zt= [yt, xt, wt] a set of equations as the ones below represent dynamic variables.

$Z_{t}=A_{1}Z_{t-1}+A_{2}Z_{t-2}+\cdots+A_{k}Z_{t-k}+\mu_{t}$ (1)

$\Delta Z_{t}=\Gamma_{1}\Delta Z_{t-1}+\Gamma_{2}\Delta Z_{t-2}+\cdots+\Gamma_{k-1}\Delta Z_{t-k-1}+\Pi Z_{t-1}+\mu_{t}$ (2)

where $\Gamma_{i}=\left( I-A_{1}-A_{2}-\cdots-A_{k} \right)(i=1,2,\ldots,k-1)$ and $\Pi=-\left( I-A_{1}-A_{2}-\cdots-A_{k} \right)$ (3)

Cointegration modelling techniques are used to understand the long-term relationship between variables. This means that if there is more than one variable, the cointegration test examines if the variables influence each other and identify which one may be leading the other variables. The cointegration technique can simplify decision-making when having multiple stocks or shares in a portfolio, as it suggests that long-term relationships are integrated between these stocks or shares into the portfolio. A number of studies, such as Kisswani and Elian (2017), Alkandari and Abul (2019) and Alshihab and Alshammari (2020), have used cointegration in order to find the long-run relationship between oil prices and the Kuwait stock market, offering up to date evidence of the value and significance of the selected econometric model. The main assumptions and limitations of the modelling framework need to be considered, as the cointegration analysis assumes that data series are non-stationary and integrated of the same order, usually integrated processes of order one or I(1) processes (Engle & Granger, 1987). The Johansen test requires large sample sizes for reliable results and assumes no structural breaks in the data (Johansen, 1991). Sample sizes are identified as a significant limitation as they can affect the power of the test, potentially leading to misidentification of cointegration relationships (Lütkepohl, 2005).

- - 1. **Causality**

The causality test is about understanding short-term relationships between two variables and their direction of influence. Granger Causality is used to examine relationships between two variables in the short-term. This statistics test determines whether changes in one variable can lead to changes in other variables. Usually, we interpret the test for two variables, X and Y as X Granger causes Y or vice versa. This tells us that the past value of X can predict Y’s current value and that Y can predict X’s current value.

The well-known Granger Causality Test (Granger, 1969) is outlined in the equations below,

$y_{t}=\alpha_{1}+\sum_{i=1}^{n} \beta_{i}x_{t-i}+\sum_{j=1}^{m} \gamma_{j}y_{t-j}+\varepsilon_{1t}$ (4)

$x_{t}=\alpha_{2}+\sum_{i=1}^{n} \theta_{i}x_{t-i}+\sum_{j=1}^{m} \delta_{j}y_{t-j}+\varepsilon_{2t}$ (5)

Where equation (4) $y_{t}$ is a dependent variable, and equation (5) $x_{t}$ is an independent variable regressed against $y_{t}$. Following Brooks (2008), Asteriou and Hall (2011) under the case that X Granger causes Y but not vice versa, then this phenomenon is called unidirectional causality. On the other hand, if the causality happens both ways, the phenomenon is called “bidirectional causality”. This research study uses unidirectional causality to analyse the impact of oil benchmarks prices due to the dual shocks on Kuwait stock market sectors by examining the short-term relationship between oil benchmarks in Kuwait stock market sectors. Therefore, the analysis of bidirectional causality is not considered the focus of interest, as the Kuwait stock market is small with no significant influence on the world’s oil benchmarks. Hence, causality modelling is used to identify short-term relationships and their direction of influence by looking at short-run movement on a continuous basis. This is of interest when looking at oil price behavior during the dual shock as it can offer insights into businesses during various economic, political or business cycles. The main objective of this study is to investigate which sectors in the Kuwait stock market are more influenced by oil price fluctuations, which is required to understand unidirectional short-term associations between sectors and the oil market. Several studies, such as Al-Shami and Ibrahim (2013), Merza and Almusawi (2016), Kisswani and Elian (2017) and Alkandari, Abul (2019), have used causality to examine the short-term relationship between oil prices and the Kuwait stock market, offering evidence of the value and significance of the selected econometric models. The modelling process considers that the Granger Causality tests assume linear relationships and stationarity of the time series data (Granger, 1969). Proper lag length selection is crucial as it can influence results (Lütkepohl, 1991). The modelling process was enhanced by implementing the VAR (p) to identify optimal lags in combination with relevant selection criteria like the AIC, SC, HQ and FPE criteria considered in cointegration and causality models. Limitations to be considered relate to the ability of the test to predict rather than establish true causality and may fail to detect non-linear dependencies (Toda & Yamamoto, 1995). Moreover, they might overlook dynamic, time-varying relationships in high-frequency data (Balcilar et al., 2010).

- - 1. **Frequency Domain Model**

The Frequency Causality Domain model by Breitung and Candelon (2006) is based on earlier work by Geweke (1982), Granger (1989) and Hosoya (1991). Breitung and Candelon (2006) developed the frequency domain causality test to determine the causality relationship for different frequencies. The model facilitates the visualisation of long, medium and short-run causality relationships between variables. A long-run causality relationship means that the resultant causality is permanent, while a short-run causality relationship is temporary. For this purpose, the Frequency Causality Domain test adds value to the proposed methodology as in combination with the Granger Causality and with the VAR model Granger (1969) and Toda-Yamamoto (1995) causality tests, it is possible to provide a richer picture of short-term dynamics in the Kuwait sectoral analysis.

Breitung and Candelon (2006) suggested a testing process based on a linear hypothesis on parameter fitting to variables using the bivariate VAR model. As a result, it is stated that the test can be generalised to examine multidimensional systems and cointegration correlation (Breitung and Candelon 2006). The Frequency Causality Domain model considers the two-dimensional vector containing $Y_{t}$ and $X_{t}$with a finite- order VAR representative of order p.

$\Theta\left( L \right)\left( \begin{aligned} &Y_{t} \\ &X_{t} \end{aligned} \right)=\left( \begin{aligned} &\Theta_{11}(L) &&\Theta_{12}(L) \\ &\Theta_{21}(L) &&\Theta_{22}(L) \end{aligned} \right)\left( \begin{aligned} &Y_{t} \\ &X_{t} \end{aligned} \right)=\varepsilon_{t}$ (6)

Where $\Theta(L)=I-\Theta_{1}L-\cdots-\Theta_{p}L_{p}$ is a 2x2 lag polynomial and $\Theta_{1},\ldots,\Theta_{p}$are 2x2 autoregressive parameter matrices, with $L^{k}X_{t}=X_{t-k}\text{ and }L^{k}Y_{t}=Y_{t-k}$ . The error vector $\varepsilon_{t}$ represents white noise with zero mean and E $\left( \varepsilon_{t}\varepsilon_{t}^{t} \right)$= Σ where Σ is positive and finite. The MA representative of the system is.

$\left( \begin{aligned} &Y_{t} \\ &X_{t} \end{aligned} \right)=\psi(L)\eta_{t}=\left( \begin{aligned} &\psi_{11}(L) &&\psi_{12}(L) \\ &\psi_{21}(L) &&\psi_{22}(L) \end{aligned} \right)\left( \begin{aligned} &\eta_{1t} \\ &\eta_{2t} \end{aligned} \right)$ (7)

With (𝐿)=Θ(𝐿)−1𝐺−1 and G is the lower triangular matrix of the Cholesky decomposition 𝐺′𝐺=Σ-1 such that 𝐸(𝜂𝑡𝜂𝑡′) =𝐼 and 𝜂𝑡=𝐺𝜀𝑡. The Causality test developed by Geweke (1982) can be written:

$M_{X\Rightarrow\gamma}(Y)=\log\left[ 1+\frac{\left| \psi_{12}\left( e^{-i\gamma} \right) \right|^{2}}{\left| \psi_{11}\left( e^{-i\gamma} \right) \right|^{2}} \right]$ (8)

In the framework, no Granger Causality from Xt to Yt with frequency y corresponds to the condition |𝜓12(𝑒−𝑖𝛾) |2=0. Breitung and Candelon’s (2006) contribution illustrates that this condition leads to

$\left| \Theta_{12}\left( e^{-i\gamma} \right) \right|=\left| \sum_{k=1}^{p} \Theta_{k,12}\cos(k\gamma) \right|-i\sum_{k=1}^{p} \Theta_{k,12}\sin(k\gamma)\mid=0$ (9)

Where, the $\Theta_{k,12}$ is the (1,2) for $\Theta_{k,}$ such that a sufficient set of conditions for no Causality is given by:

$\sum_{k=1}^{p} \Theta_{k,12}\cos(k\gamma)\mid=0\text{ and }\sum_{k=1}^{p} \Theta_{k,12}\sin(k\gamma)=0$(10)

The frequency domain causality test was applied to understand the dynamic short-run relationship between oil benchmarks in Kuwait’s stock market sectors. Hence, as in the literature, several studies have investigated the relationship between macroeconomic variables such as oil prices and the stock market using the Frequency Causality Domain model. For example, Algahtani et al. (2019) used the frequency domain Causality method to examine oil price volatility on the GCC countries’ sectoral returns. The frequency domain causality method shows that contagion and interdependency effects affect the type of causality in the short and long run. Their findings illustrate that all sectors are found to be interdependent with oil price volatility, except the energy and transportation sectors.

Additionally, using the Frequency Causality Domain model, Abdulrazzaq et al. (2018) have investigated the relationship between macroeconomic variables such as oil prices and the stock market. Furthermore, Alshammari et al. (2020) used the Frequency Causality Domain model to examine the impact of the exchange rate, oil and gold prices on the Kuwait stock market for the time period from January 2, 1996, to September 28, 2017. Likewise, Almassari et al. (2020) used the Frequency Causality Domain model to test the relationship between Kuwait’s economic growth and financial development. The Breitung and Candelon’s Frequency Domain Model relies on the stationarity of the series and proper lag structure specification. It also assumes that causal relationships vary by frequency instead of being constant over time (Breitung & Candelon, 2006). The model can be sensitive to specification errors leading to complicating interpretation or outcomes (Wang et al., 2020). Moreover, the methodology might not account for non-cyclic relationships or those outside the specified frequency ranges (Fan & Yao, 2003).

**3.2.5 Justification for Model Selection and Robustness Checks**

The paper acknowledges the critical importance of robustness in time series analysis to ensure the stability and accuracy of results. The implemented models align with previous research in the region and, as per guidelines offered by the conducted literature review (e.g., Alshihab & Alshammari, 2020; Kisswani & Elian, 2017), ensure methodological consistency and facilitate meaningful comparisons. Although more advanced techniques, such as machine learning or non-linear models, exist, classical cointegration and causality approaches are preferred due to their efficiency and strong theoretical foundations. This was needed to provide clear insights to policy-makers and investors; in contexts where oil leads economic activity, simpler, clearer models are needed, as the purpose is to provide solid results that enable research-informed decision-making without introducing unnecessary complexity (Asteriou & Hall, 2011; Toda & Yamamoto, 1995).

Traditional robustness checks that include testing the residuals for serial correlation and heteroskedasticity do not apply to the selected models as they are implemented using the variables in levels, and the outcomes of standard robustness checks will lead to invalid results, which are also documented in the literature. For instance, Alshihab and AlShammari (2020) applied Johansen Cointegration and Granger Causality methods, supported by ADF and PP tests, to explore oil–stock relationships in Kuwait without reporting supplementary robustness tests for residuals, as they are not valid for the selected models. Similarly, Kisswani and Elian (2017) adopted Johansen Cointegration and Granger Causality in a non-linear ARDL framework and did not integrate residual checks. Prabheesh et al. (2020) used ADF and PP tests along with Johansen’s method, but also refrained from further validation. These decisions are consistent with a pattern observed in many oil-market studies that acknowledge the limitations of some time series models regarding robustness checks that are addressed with the implementation of a variety of econometric models to ensure consistency and robustness while prioritising clarity and model interpretability, particularly when the research context involves data constraints or focused sectoral analysis. Alsamman and Akkas (2022) followed a similar path, using Engle-Granger cointegration and Granger Causality models to study the economic impact of COVID-19 and oil shocks on GCC countries without robustness extensions due to the outlined limitations. In broader global contexts, Huang et al. (1996) and Jimenez-Rodriguez and Sanchez (2005) also employed Granger Causality and Cointegration analyses without incorporating parameter stability, reflecting a reliance on classical inference standards. Additional studies support this trend, like Arouri et al. (2011), who examined oil stock return and volatility spillovers in GCC economies using Johansen and Granger frameworks without reporting diagnostic checks for VAR stability; Maghyereh et al. (2017) focused on volatility spillovers using Johansen Cointegration and Causality, again without validating spillover dynamics with alternate specifications. Moreover, Trabelsi (2017) and Sim and Zhou (2015) also relied on cointegration and quantile-specific Granger Causality approaches, respectively, without conducting further robustness tests. Beyond the Gulf region, initial work by Imarhiagbe (2010), Serletis and Banack (1990), Quan (1992), and Schwartz and Szakmary (1994) applied similar methods in analysing petroleum markets and futures linkages without robustness testing. Furthermore, Dwipraptono (2007), Fernando and Sharpe (2015), Tran and Dao (2023), and Dai et al. (2014) apply cointegration and causality models without using robustness checks, especially when results across core tests were consistent, as researchers acknowledge the complexities of time series analysis that in the context of the used models requires a different approach than the “traditional” tests for robustness.

Finally, these examples illustrate that the absence of robustness checks does not indicate methodological weakness. However, in order to strengthen this research study and to provide a robust framework, this paper introduces a combination of theoretically justified econometric models; this study addresses the limitations of the reviewed literature that are also identified as a novel contribution as the implementation of different econometric models helps in terms of ensuring validity and robustness of the result when traditional tests on residuals are not suitable to the modelling process.
