# Supplementary figures and images for: Sectoral sensitivity of the Kuwait stock market to a dual shock
Source: PLoS One. 2025 Sep 24;20(9):e0331384. doi: 10.1371/journal.pone.0331384 (PMC12459840; doi:10.1371/journal.pone.0331384)

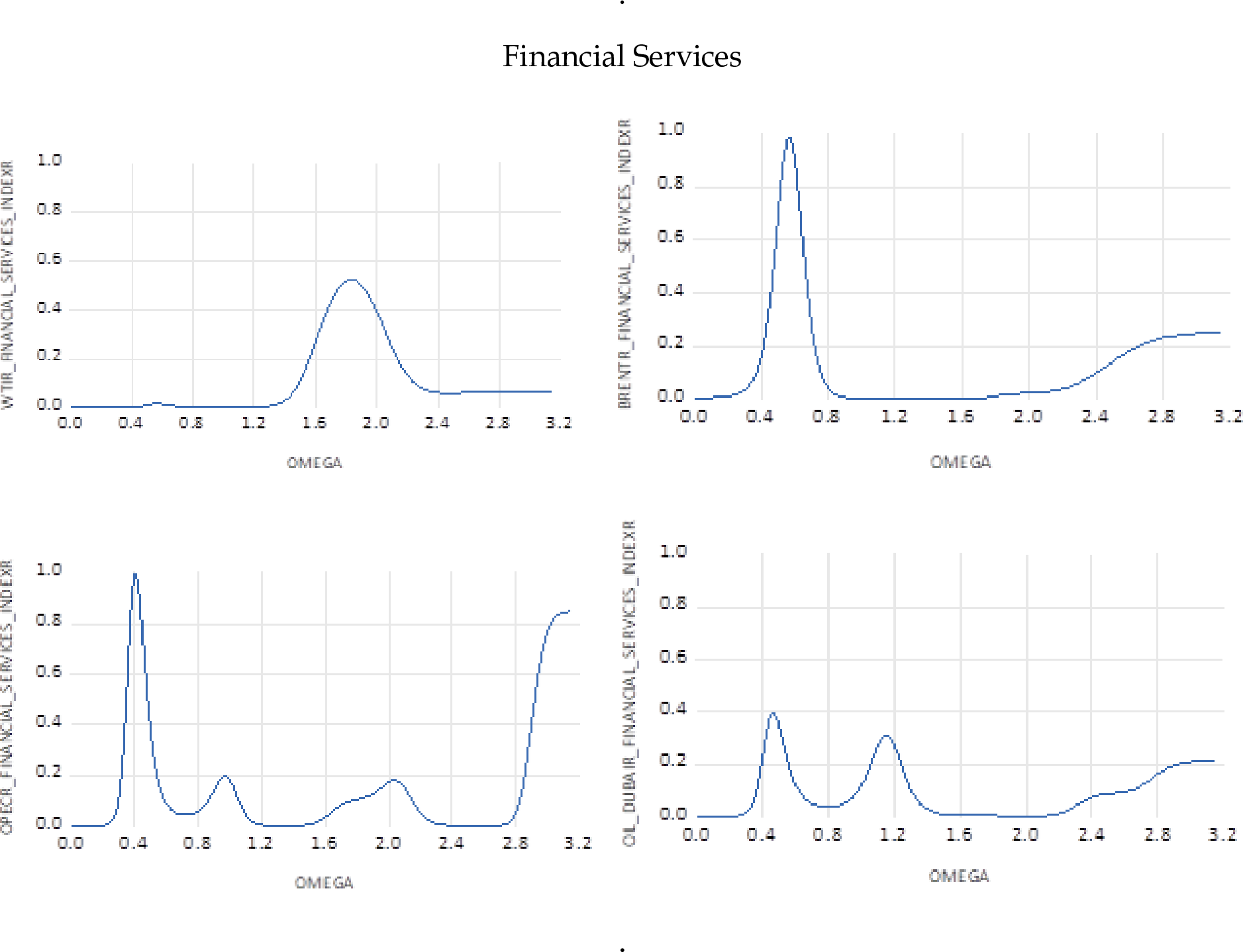

Supplement: S3 Table — (DOCX) [file pone.0331384.s004.tif]

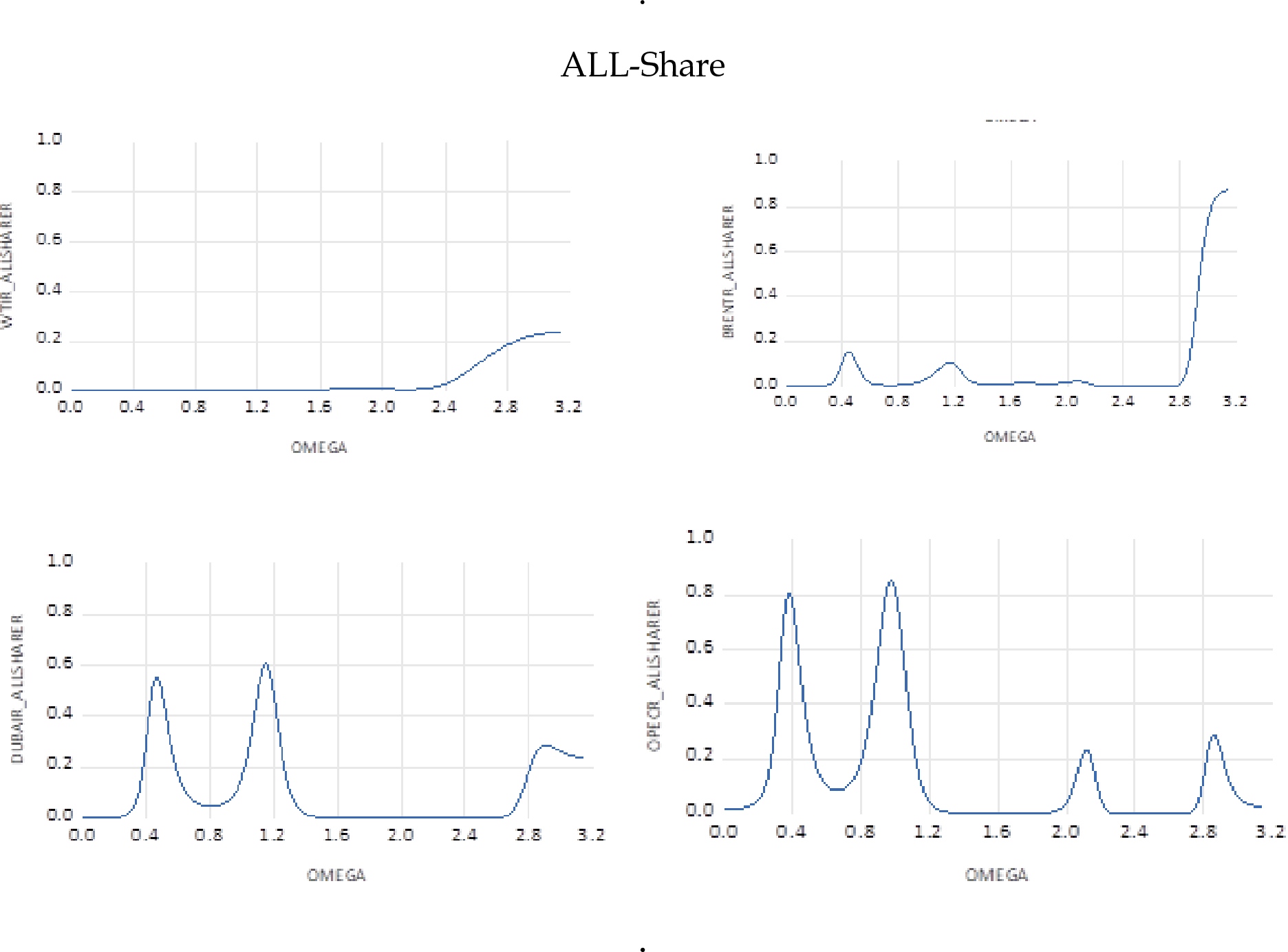

Supplement: S1 Fig — (TIF) [file pone.0331384.s008.tif]

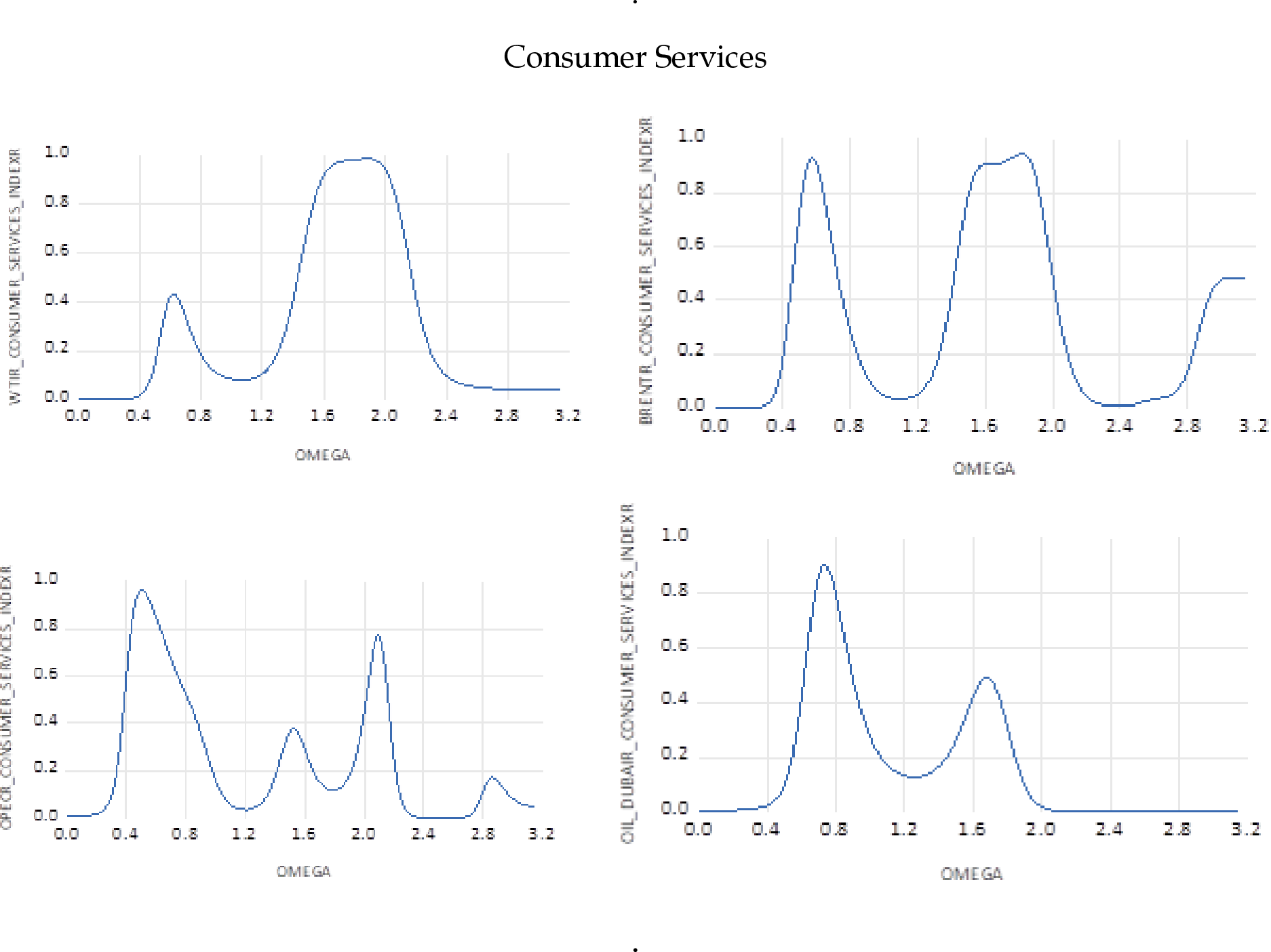

Supplement: S2 Fig — (TIF) [file pone.0331384.s009.tif]

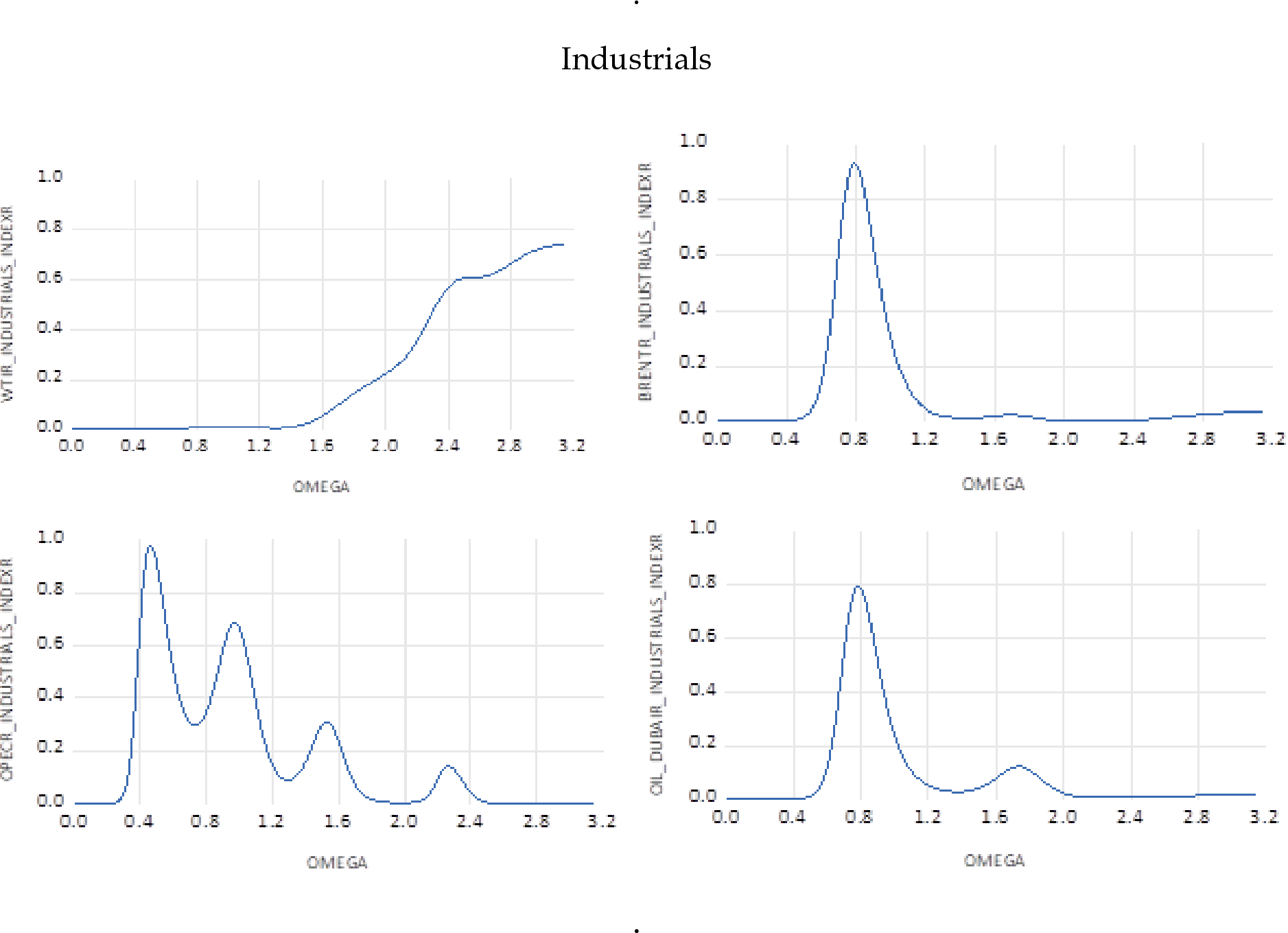

Supplement: S4 Fig — (TIF) [file pone.0331384.s011.tif]

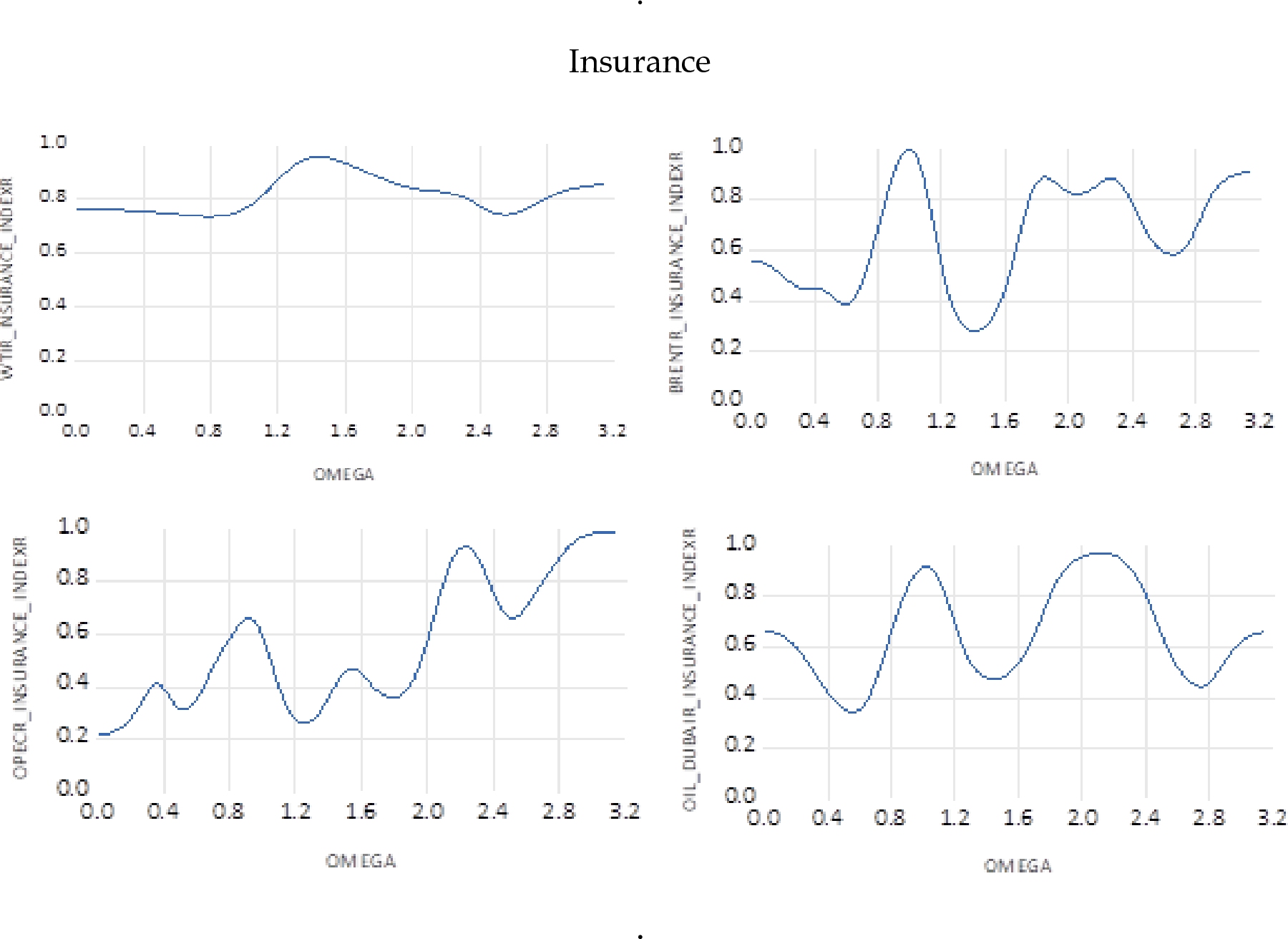

Supplement: S5 Fig — (TIF) [file pone.0331384.s012.tif]

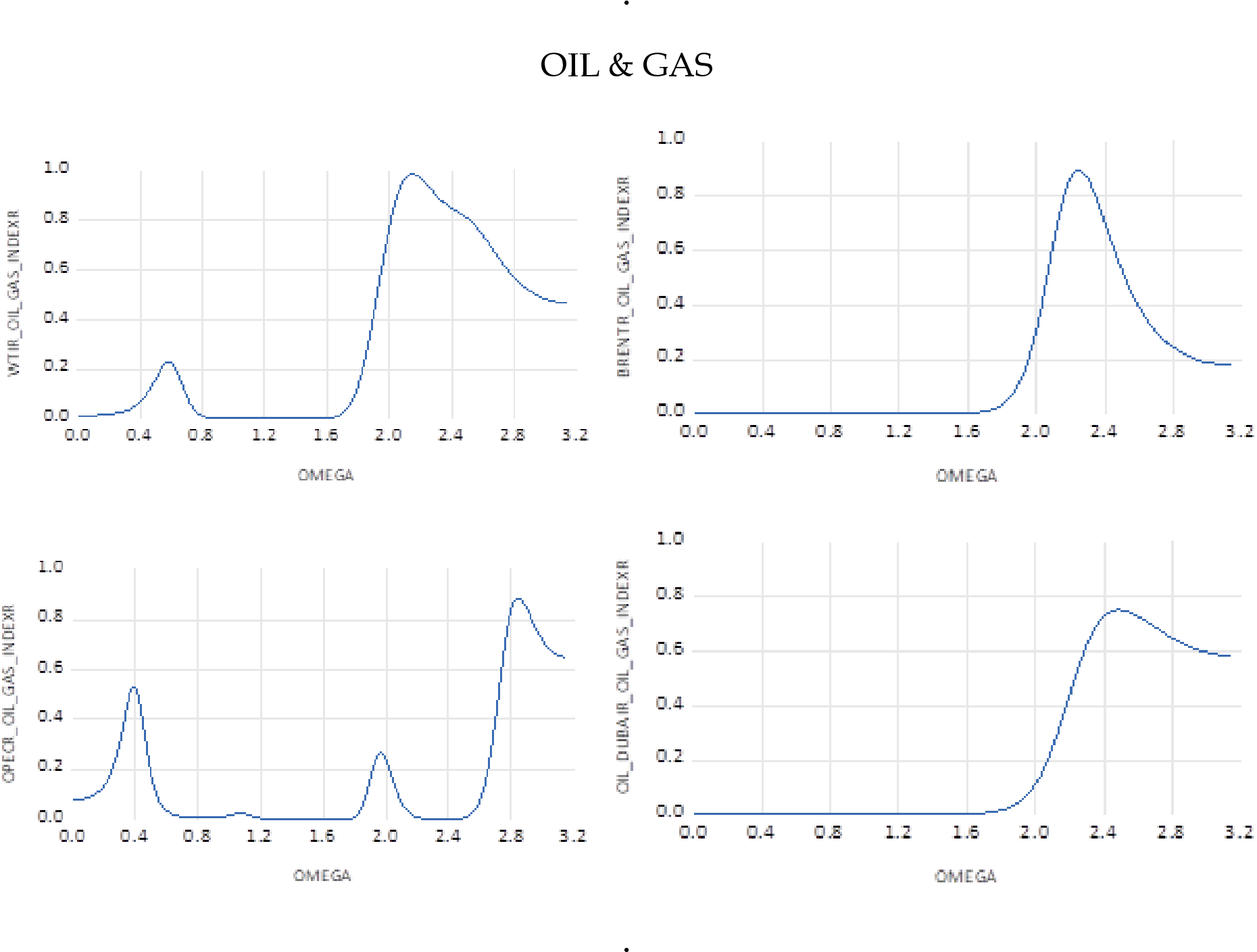

Supplement: S6 Fig — (TIF) [file pone.0331384.s013.tif]

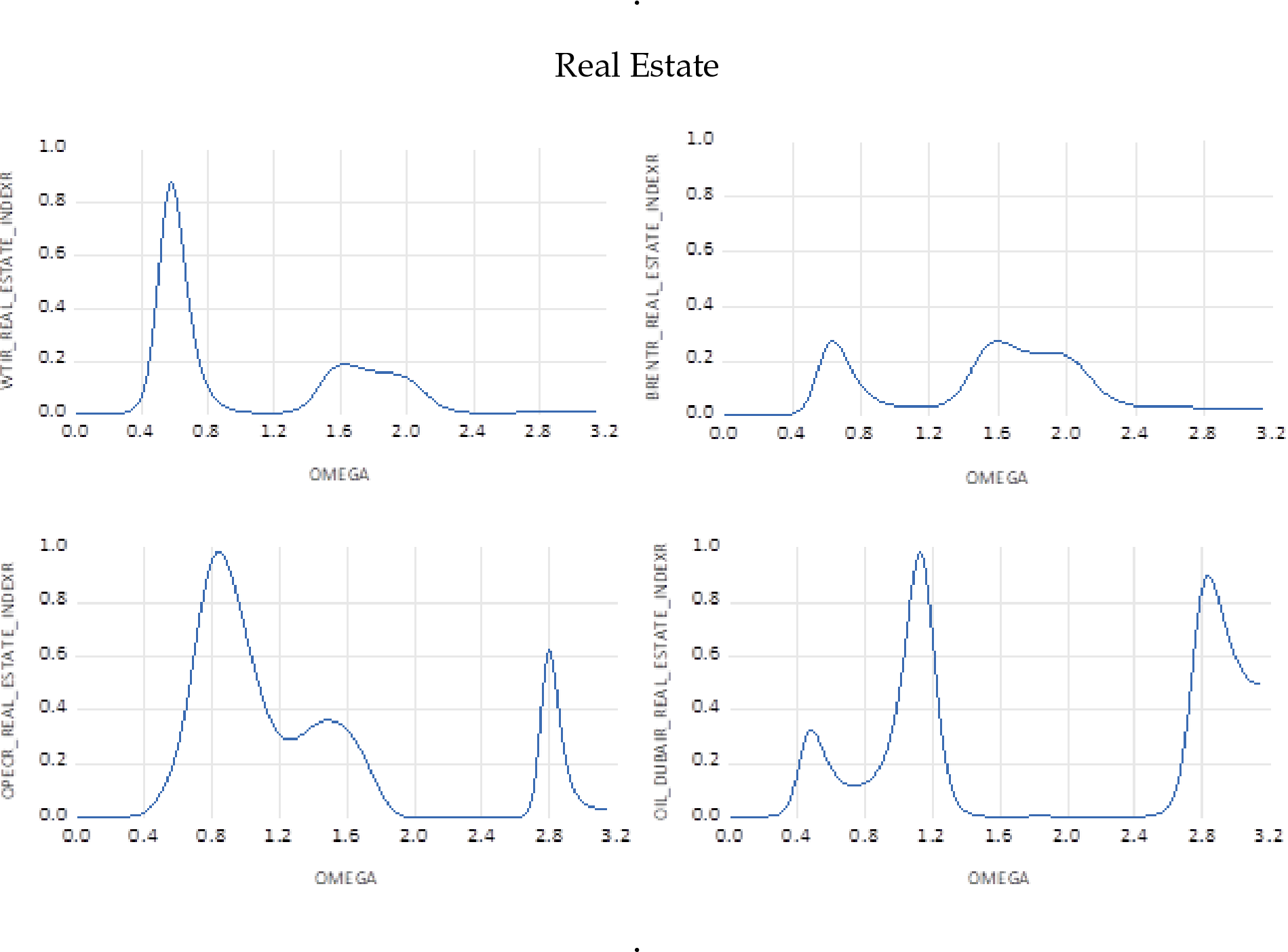

Supplement: S7 Fig — (TIF) [file pone.0331384.s014.tif]

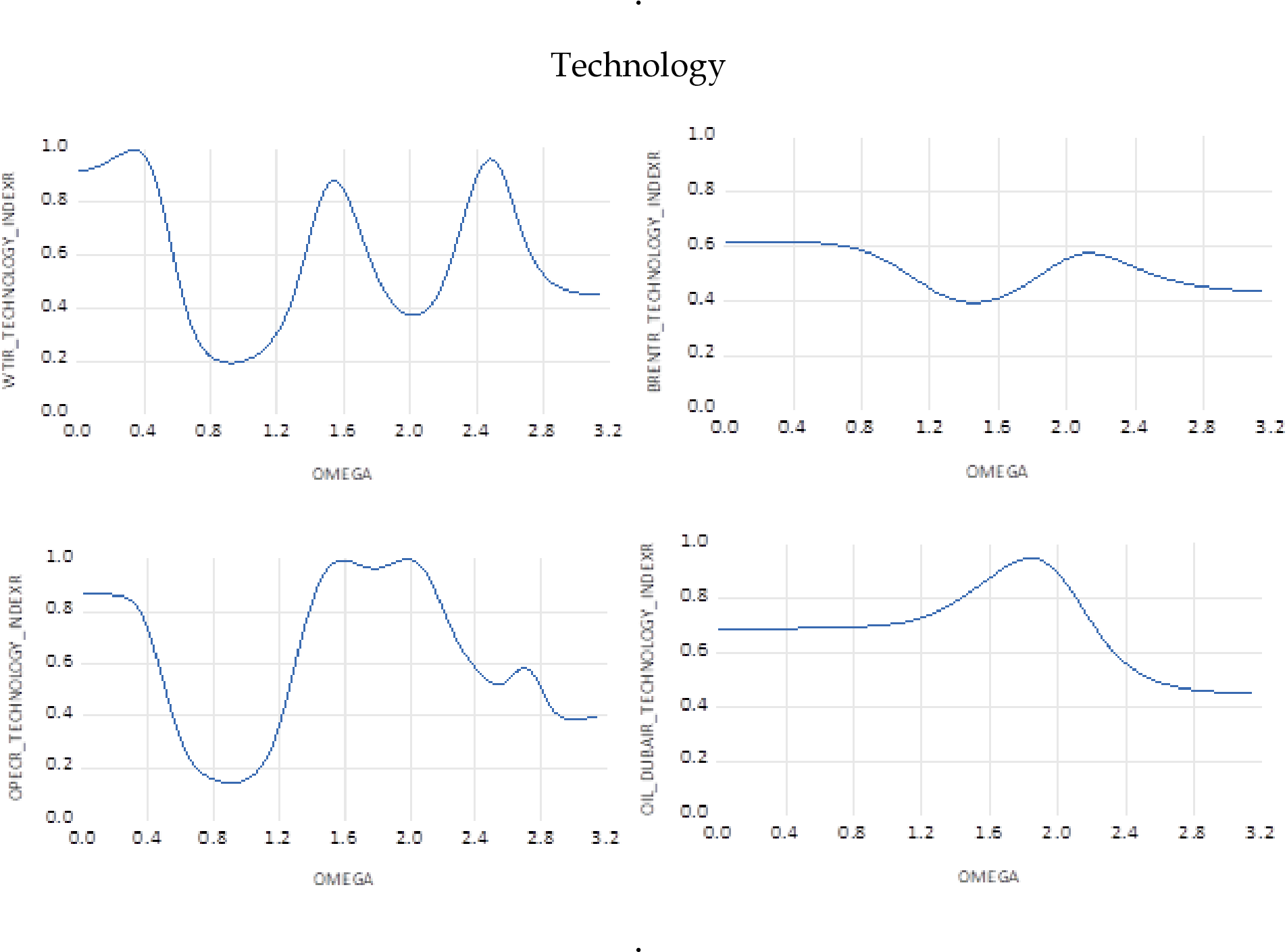

Supplement: S8 Fig — (TIF) [file pone.0331384.s015.tif]

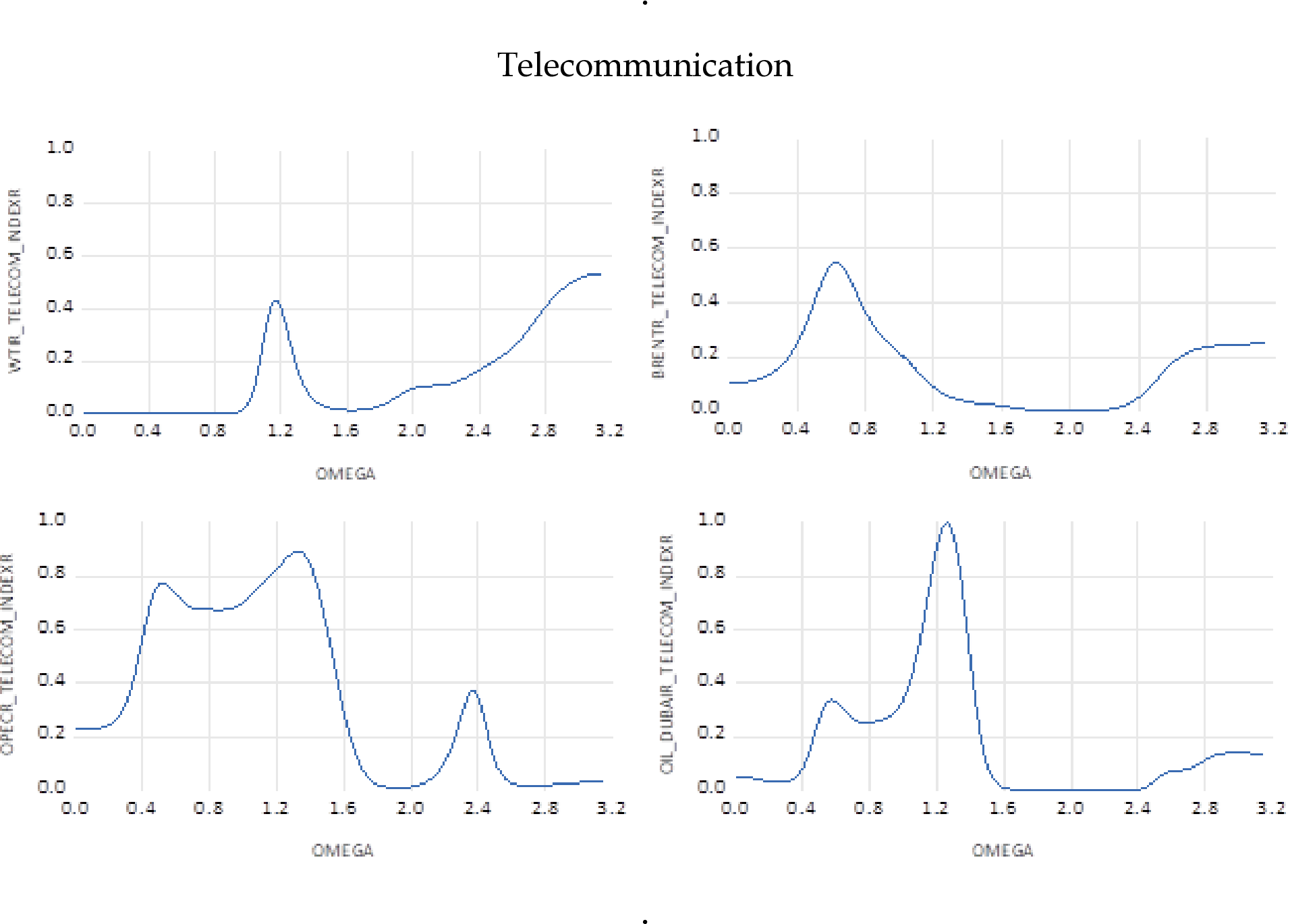

Supplement: S9 Fig — (TIF) [file pone.0331384.s016.tif]
