# Supplementary material for: Sectoral sensitivity of the Kuwait stock market to a dual shock
Source: PLoS One. 2025 Sep 24;20(9):e0331384. doi: 10.1371/journal.pone.0331384 (PMC12459840; doi:10.1371/journal.pone.0331384)
Supplement: S4 Table — (DOCX) [file pone.0331384.s005.docx]

|  | **Returns** | | |  | **Price** | | |  |
| --- | --- | --- | --- | --- | --- | --- | --- | --- |
| **OIL** | **ADF** | **PP** | **KPSS*** | **lags** | **ADF** | **PP** | **KPSS*** | **lags** |
| **Brent** | -38.91727 (0.0000) | -38.93839 (0.0000) | 0.081250 (0.739000) | 0 | -1.114668 (0.7121) | -1.239613 (0.6592) | 0.918285 | 1 |
| **Dubai** | -40.48919 (0.0000) | -40.52904  (0.0000) | 0.085318 (0.739000) | 0 | -1.279019 (0.6413) | -1.290812 (0.6359) | 1.076009 | 1 |
| **OPEC** | -38.31866  (0.0000) | -39.18645 (0.0000) | 0.073476 (0.739000) | 0 | -1.056800 (0.7345) | -1.270382 (0.6453) | 1.060593 | 2 |
| **WTI** | -38.97423  (0.0000) | -39.03769 (0.0000) | 0.068929 (0.739000) | 0 | -1.237253  (0.6602) | -1.701877 (0.4302) | 0.883950 | 4 |

S4 Table. Stationarity Test Oil Benchmarks

***Note:*** *the p-values are shown in parentheses; there is no p-value for KPSS; therefore, the 1% significance level was considered for the test at a value of 0.739000. The main indices for the major oil Benchmarks are presented, and the results for the three stationarity tests with p-values in brackets and the number of required lags to estimate the tests are reported in the table. Source: Data Stream (2022)*
