# Supplementary material for: Sectoral sensitivity of the Kuwait stock market to a dual shock
Source: PLoS One. 2025 Sep 24;20(9):e0331384. doi: 10.1371/journal.pone.0331384 (PMC12459840; doi:10.1371/journal.pone.0331384)
Supplement: S5 Table — (DOCX) [file pone.0331384.s006.docx]

| **Cointegration test** | | | | | | | | | | | | | |
| --- | --- | --- | --- | --- | --- | --- | --- | --- | --- | --- | --- | --- | --- |
|  | **All share** | **Banks** | **REAL-ESTATE** | **Financial Services** | **Telecom** | **Consumer Services** | **OIL & Gas** | **Healthcare** | **Insurance** | **Basic Materials** | **Consumer Goods** | **Industrials** | **Technology** |
| **WTI** | **YES**   (0.0052)** | **YES**   (0.0354)*** | **YES**   (0.0073) ** | **YES**   (0.0175)*** | **YES**   (0.0169)*** | **NO**   (0.1602) | **YES**   (0.0009)* | **YES**   (0.0252)*** | **YES**  (0.0047)** | **YES**  (0.0000)* | **NO**  (0.1147) | **YES**  (0.0000)* | **YES**   (0.0661)*** |
| **Brent** | **NO**   (0.1426) | **NO**  (0.7280) | **YES**   (0.0987)*** | **NO**   (0.3938) | **YES**   (0.0359)*** | **NO**   (0.3345) | **YES**   (0.0065)** | **YES**   (0.0511)*** | **YES** (0.0896)*** | **YES**  (0.0075)** | **NO**  (0.1910) | **YES**  (0.0486)*** | **YES**   (0.0800)*** |
| **OPEC** | **NO**   (0.1793) | **NO**  (0.1924) | **NO**   (0.1378) | **NO**   (0.5122) | **YES**   (0.0344)*** | **NO**   (0.4286) | **YES**   (0.0070)** | **YES**   (0.0576)*** | **NO**   (0.1263) | **YES** (0.0161)*** | **NO**  (0.2026) | **NO**  (0.1019) | **YES**   (0.0798)*** |
| **DUBAI** | **NO**   (0.1126) | **NO**  (0.2560) | **NO**   (0.1196) | **NO**   (0.4231) | **YES**   (0.0321)*** | **NO**   (0.4296) | **YES**   (0.0046)** | **YES**   (0.0498)*** | **YES** (0.0915)*** | **YES**  (0.0029)** | **NO**  (0.1701) | **YES**  (0.0280)*** | **YES**   (0.0752)*** |

S5 Table Cointegration Findings

*The superscripts (*), (**) and (***) indicate that the parameter is significant at 1%, 5%, and 10% level respectively. Source: Data Stream (2023)*
