# Supplementary material for: Sectoral sensitivity of the Kuwait stock market to a dual shock
Source: PLoS One. 2025 Sep 24;20(9):e0331384. doi: 10.1371/journal.pone.0331384 (PMC12459840; doi:10.1371/journal.pone.0331384)
Supplement: S6 Table — (DOCX) [file pone.0331384.s007.docx]

| **Causality test** | | | | | | | | | | | | | |
| --- | --- | --- | --- | --- | --- | --- | --- | --- | --- | --- | --- | --- | --- |
|  | **All share** | **Banks** | **REAL-ESTATE** | **Financial Services** | **Telecom** | **Consumer Services** | **OIL & Gas** | **Healthcare** | **Insurance** | **Basic Materials** | **Consumer Goods** | **Industrials** | **Technology** |
| **WTI** | **NO**  (0.2146) | **NO** (0.5247) | **YES**  (2.E-05)* | **YES**  (0.0049)** | **YES**  (0.0967)*** | **NO**  (0.9717) | **NO**  (0.2603) | **NO**  (0.9993) | **NO**  (0.3985) | **YES**  (0.0026)** | **YES**  (0.0123)*** | **NO**  (0.1976) | **NO**  (0.9757) |
| **Brent** | **YES**  (0.0305)*** | **YES**  (0.0604)*** | **YES**  (5.E-05)* | **YES**  (0.0022)** | **NO**  (0.4238) | **NO**  (0.2115) | **NO**  (0.7876) | **NO**  (0.8691) | **NO**  (0.5364) | **YES**  (0.0007)* | **YES**  (0.0068)** | **NO**  (0.1727) | **NO**  (0.8099) |
| **OPEC** | **YES**  (0.0045)** | **YES**  (0.0018)** | **YES**  (8.E-05)* | **YES**  (0.0402)*** | **NO**  (0.6932) | **NO**  (0.2908) | **YES**  (0.0218)*** | **NO**  (0.9925) | **NO**  (0.7161) | **YES**  (0.0014)** | **NO**  (0.4940) | **YES**  (0.0528)*** | **NO**  (0.9163) |
| **DUBAI** | **YES**  (0.0043)** | **YES**  (0.0058)** | **YES**  (0.0002)* | **YES**  (0.0025)** | **NO**  (0.3258) | **YES**  (0.0555)*** | **NO**  (0.9203) | **NO**  (0.0904)*** | **NO**  (0.7466) | **YES**  (6.E-05)* | **YES**  (0.0158)*** | **NO**  (0.1093) | **NO**  (0.6964) |

S6 Table Causality Findings

*The superscripts (*), (**) and (***) indicate that the parameter is significant at 1%, 5%, and 10% level respectively. Source: Data Stream (2023)*
