# Supplementary material for: Sectoral sensitivity of the Kuwait stock market to a dual shock
Source: PLoS One. 2025 Sep 24;20(9):e0331384. doi: 10.1371/journal.pone.0331384 (PMC12459840; doi:10.1371/journal.pone.0331384)
Supplement: Attachment — Submitted filename: PONE-D-23-38848_RefreeReport.pdf [file pone.0331384.s017.pdf]

Referee report

PONE-D-23-38848

"Sectoral Sensitivity of the Kuwait Stock Market to a Dual Shock"

The research focuses on the sensitivity of different sectors within Kuwait's stock market to two major shocks in 2020: the COVID-19 pandemic and the oil price instability. It investigates how various sectors of Kuwait's stock market responded to these dual shocks using traditional Cointegration and Causality models, as well as the frequency domain model by Breitung and Candelon (2006). Utilizing daily returns data from December 31, 2015, to February 23, 2022, authors examine the long- and short-term dynamics between four oil benchmarks (Brent, Dubai, OPEC, and WTI) and Kuwait's stock market sectors during the 2020 crisis.

While the exact magnitudes of the key findings are absent in both the abstract and the introduction, the authors argue that their findings reveal a significant impact of these oil benchmarks on Kuwait's stock market sectors. I find the study interesting due to its focus on the stock market of a developed oil-exporting country. However, I observed that the authors missed the opportunity to present their findings more coherently and methodologically in the current paper. Therefore, I would like to suggest a few issues to address in the revised version of this paper:

1. Several crucial points are missing in the abstract, and as a reader, I would like to see the following addressed:

a. While the abstract mentions the dual shocks, it lacks a detailed explanation and the magnitudes of how each shock specifically affected the Kuwaiti economy and stock market.

b. The abstract does not specify which sectors of the stock market were most affected by the dual shocks. Identifying these sectors would enhance the reader's understanding of sectoral sensitivity.

c. Readers may struggle to separate the potential policy recommendations or strategies that could be derived from the research findings. Additionally, including a brief comparison of Kuwait's

experience with that of other oil-exporting economies could provide a broader perspective on the generalizability of the findings.

2. The first two paragraphs on page 1 correctly highlight the dominant focus of existing research on developed economies, specifically OECD countries, and identify the gap in literature concerning oil-exporting economies, particularly the Gulf Cooperation Council (GCC) economies and Kuwait. However, the background information on dual shocks, sectoral breakdown, and the linkage between the global health crisis of 2020 and its significant impact on Kuwait's macroeconomy and stock markets is necessary to fully understand the context and importance of the study, which is not explicitly and comprehensively presented in the introduction. I understand that those information is separately presented in the subsequent two sections but the should be move here in the starting section of the introduction.

On page 1, the first line of paragraph 2 in the introduction mentions the dual shocks of COVID-19 and the oil price war but does not provide enough detail on how these events unfolded and their specific impacts on the global and Kuwaiti economies.

3. Like I mentioned, to address the background information consistently, I suggest moving the sections “2. Dual Shock – Insights from Global Impact towards Sectoral Level” and “Insights at the Global Markets Level” to page one after the first paragraph, incorporating the necessary information on the dual shocks and sectoral breakdown. These two sections are unnecessarily lengthy and could be significantly shortened. Additionally, the authors do not explicitly state the specific motivation for choosing Kuwait as the focus of the study. While the text mentions the global health crisis and the oil price war, it does not clearly connect these events to the specific need to study Kuwait’s stock market sectors.

4. I suggest that the authors provide a more detailed elaboration on Kuwait's economic structure, its dependence on oil, and the specific vulnerabilities it faced during the dual shocks. While the introduction acknowledges a scarcity of research on sectoral performance in GCC economies, it

lacks specificity regarding the findings of existing studies and how this study intends to address this gap.

5. It is probably better to introduce the key findings early in the introduction, followed by their implications for potential policy recommendations arising from the study. The discussion should emphasize how understanding sectoral sensitivity to shocks can inform policies aimed at economic diversification and risk mitigation in oil-dependent economies. Furthermore, the study should outline its contributions to the existing literature on the impact of dual shocks on stock markets, highlighting new insights into the sectoral performance of Kuwait's stock market.

6. Prior to examining into the data and methodology section, I suggest the authors develop a section outlining their research questions and potential hypotheses for exploration in this paper. The hypothesis section is currently absent.

7. I suggest that the authors briefly provide the rationale behind their decision to begin the analysis from December 31, 2015, focusing on the Kuwait Weighted Market Index, sectoral indices, and crude oil benchmarks. It is unclear why the authors omitted data preceding 2015, especially when such observations could readily be retrieved from DataStream.

They should discuss any significant economic events or market conditions around that time that justify this starting point. Additionally, they should explicitly connect the selection of these specific indices and benchmarks to the research objectives, explaining how these choices will facilitate understanding of sectoral sensitivity to the dual shocks of COVID-19 and the oil price war. The section should also include a key summary statistics table, providing essential insights into the summary statistics.

8. On page 7, the authors mention consideration of three well-known stationarity tests: the Augmented Dickey-Fuller (ADF) test (1979), Phillips-Perron (PP) test (1988), and Kwiatkowski-Phillips-Schmidt-Shin (KPSS) test (1992). I suggest they present the key insights of the stationarity tests in this section, with detailed tables related to these tests placed in the appendix instead of the table currently located on page 18.

9. The analysis of the three subsections “3.1.1 Cointegration,” “3.1.2 Causality,” and “3.1.3 Frequency Domain Model” should be more closely aligned with the research questions and hypotheses of the authors. I suggest they provide a comprehensive discussion of these sections in relation to their research hypotheses, and consider condensing them for clarity.

10. The methodology employed includes traditional Cointegration and Causality models, alongside the dynamic causal modeling of short-run dynamics introduced by Breitung and Candelon (2006). However, the section lacks mention of any assumptions or limitations associated with these models, including the frequency domain model. Addressing these would enhance transparency in the research methodology.

As a reader, I am more interested to understand how the use of Cointegration and Causality models, as well as the Breitung and Candelon's frequency domain model, represents a novel approach to data analysis compared to more advanced methodologies currently employed in similar research.

11. Figure 1 should not serve as the starting point for the “Core Research Findings.” Instead, I suggest integrating this figure into the data section, where the key summary statistics table is currently absent. Similarly, Figure 2 and its descriptive content should be relocated to the data section, with Figure 2 ideally placed in the appendix.

12. Pages 19 to 20 present the key results of the Cointegration & Causality Findings. However, the explanations provided in this section are purely of a statistical nature and lack a connection to the research questions and hypotheses. It is essential to justify these findings from the perspective of economic and financial theories, which is currently absent. Also, I find this section is bit incomplete.

13. The paper lacks robustness analysis, making it impossible to assess the validity of the results. It remains unclear how sensitive these findings are to changes in data points or subsections of the data. Furthermore, the portfolio and investment decision implications of these findings are not discussed. Without clear portfolio implications stemming from robust findings, the authors should refrain from claiming scientific significance in contemporary literature.

I hope the authors find these suggestions helpful for revising their paper.

Good luck with your revisions.
