# Supplementary material for: Sectoral sensitivity of the Kuwait stock market to a dual shock
Source: PLoS One. 2025 Sep 24;20(9):e0331384. doi: 10.1371/journal.pone.0331384 (PMC12459840; doi:10.1371/journal.pone.0331384)
Supplement: Attachment — Submitted filename: PONE-D-23-38848R1_Referee report.pdf [file pone.0331384.s019.pdf]

Referee report

PONE-D-23-38848R1

Sectoral Sensitivity of the Kuwait Stock Market to a Dual Shock

I acknowledge the authors' efforts in addressing most of my previous comments. However, the revised manuscript has introduced new issues regarding organization, redundancy, overly lengthy and mechanical explanations, and repetitive content across several sections, making it difficult to identify key information. For instance, the abstract could be more concise without losing essential details. A clearer opening sentence might be: "*This study examines Kuwait's stock market sectors' response to the dual shock, employing traditional cointegration and causality models alongside dynamic causal modeling to analyze short-run dynamics through frequency domain analysis.*" Beyond this, I find only four sentences are necessary (and sufficient) for the abstract.

Below are additional points that may help improve the manuscript:

1. Overall, the manuscript is not yet at the level expected for a scientific journal publication. The abstract and introduction are particularly critical, yet I find it challenging to distinctly and consistently convey the following:

- What the research question is (looks like this appears acceptable now) and the related hypotheses to be tested.
- Its significance and why it's important (considering Kuwait, the Middle Eastern economy, and potentially the global economy).
- The methodology and rationale (how and why the chosen approach is appropriate and why did the author choose very basic time series approaches instead of frequently used advanced techniques, why do the robustness tests are not necessary for the benchmark results).
- Key findings, novelty, and contributions (what you discovered, what is new, and how this advances the field).

I request the authors to ensure these elements are explicitly and clearly presented in both the abstract and introduction.

2. Sections 1.1–1.3 and Section 2 could be easily combined into just one or two pages without losing essential content.

3. Also, the current subdivision of the introduction section does not follow standard academic conventions. I recommend rewriting this section to better align with the coherent structure I outlined in my previous comments (point 1).
4. Section 3 contains explanations and contents that could be moved to an appendix (e.g., methodological details) since it does not add any new insight to the readers and to the core narrative. Section 3.1.3 (Descriptive Analysis) should instead follow immediately after the data description for better flow.
5. Tables 2 and 3 do not appear to provide significant insights beyond what is already stated in the text. The accompanying notes under each Tables merely repeat mechanical explanations found in the main body, making them redundant.
6. Please remember, "***The quality of a paper is monotonically decreasing in length – shorter is better, everything else held constant.***" I strongly recommend tightening all sections to focus on key points efficiently.
7. The section titled "***3.1.1 The Research Question and Hypothesis***" needs to be revised, as the key hypotheses and the final description do not align well with the introduction. Additionally, there is unnecessary repetition of content that was already covered in the introduction.

8. Based on my understanding of the introduction and hypotheses, the sections *3.2.1 Stationarity, 3.2.2 Cointegration, and 3.2.3 Causality* should either be moved to the appendix or revised to better align with the research questions/hypothesis. You need to explain how each of these **very basic time series concepts** is connected to the hypotheses and whether they support or refute them.

9. I noticed that there is a contrast between your findings and the previous studies discussed in ***section 5, "Critical Discussions***." It is important to explain why your findings differ from previous research and highlight any new insights that support the conclusions of earlier studies.

10. The revised ***manuscript with track changes*** does not adhere to the standard manuscript submission style. Please ensure that this is addressed in future submissions.
