# Supplementary material for: Sectoral sensitivity of the Kuwait stock market to a dual shock
Source: PLoS One. 2025 Sep 24;20(9):e0331384. doi: 10.1371/journal.pone.0331384 (PMC12459840; doi:10.1371/journal.pone.0331384)
Supplement: Attachment — Submitted filename: Referee report_Response.docx [file pone.0331384.s018.docx]

Public Library of science (PLOS)

Subject: Original manuscript resubmission

Dear PlosOne Editors,

We would like to thank you for all the comments and feedback on our manuscript. We have completed all requested changes and have developed a comprehensive report to support our manuscript resubmission.

The core points to consider include:

1. Updated manuscript with track changes to ensure that you are able to identify all amendments and additional information.
2. Updated manuscript without track changes.
3. Response letter with all comments addressed and full explanations provided that you can find below.

We remain at your disposal for any further comments or clarifications that might be needed.

Yours sincerely,

Talal Alotaibi

Referee report

PONE-D-23-38848

“Sectoral Sensitivity of the Kuwait Stock Market to a Dual Shock”

The research focuses on the sensitivity of different sectors within Kuwait’s stock market to two major shocks in 2020: the COVID-19 pandemic and the oil price instability. It investigates how various sectors of Kuwait’s stock market responded to these dual shocks using traditional Cointegration and Causality models, as well as the frequency domain model by Breitung and Candelon (2006). Utilising daily returns data from December 31, 2015, to February 23, 2022, authors examine the long- and short-term dynamics between four oil benchmarks (Brent, Dubai, OPEC, and WTI) and Kuwait’s stock market sectors during the 2020 crisis.

While the exact magnitudes of the key findings are absent in both the abstract and the introduction, the authors argue that their findings reveal a significant impact of these oil benchmarks on Kuwait’s stock market sectors. I find the study interesting due to its focus on the stock market of a developed oil-exporting country. However, I observed that the authors missed the opportunity to present their findings more coherently and methodologically in the current paper. Therefore, I would like to suggest a few issues to address in the revised version of this paper:

**1. Several crucial points are missing in the abstract, and as a reader, I would like to see the following addressed:**

**a. While the abstract mentions the dual shocks, it lacks a detailed explanation and the magnitudes of how each shock specifically affected the Kuwaiti economy and stock market.**

The dual shock of 2020, identified as the global health pandemic (COVID-19) and the price war between Russia and the Kingdom of Saudi Arabia had a dual effect on the Kuwaiti economy. The COVID-19 health crisis grew into an economic and financial dimension of international proportions as the virus spread worldwide. Some economies introduced restrictions on economic activities as they sought to control the spread of COVID-19. Introduced health measures led to a sharp decrease in consumption and economic production for some businesses and industries, with significant implications for global supply chains. The disruptions on economic activity also affected the dynamics of the economic and financial systems.Alongside this, the oil price war between the Kingdom of Saudi Arabia and Russia created further pressure on oil exporting countries and their economic systems. The global financial market was negatively impacted due to the reduction of oil demand in the commodity markets, especially in oil prices, tat experienced a decline in prices by more than 20 percent in one day.

**b. The abstract does not specify which sectors of the stock market were most affected by the dual shocks. Identifying these sectors would enhance the reader’s understanding of sectoral sensitivity.**

The research findings showed that the dual shock negatively affected consumer services, industrial, and basic materials sectors by registering a price drop of approximately 30 percent across the mentioned sectors. The research findings signal the sensitivities of Kuwait’s specific sectors to oil price fluctuations and underline the deep influence of world oil dynamics on the functioning of the country’s monolithic economic system heavily dependent on oil exports.

**c. Readers may struggle to separate the potential policy recommendations or strategies that could be derived from the research findings. Additionally, including a brief comparison of Kuwait’s experience with that of other oil-exporting economies could provide a broader perspective on the generalizability of the findings.**

The research findings offer evidence of a significant impact of the four selected oil benchmarks (Brent, Dubai, OPEC and WTI) on the behaviour of Kuwait’s stock market sectors, highlighting the country’s heavy dependence on its oil mono-economy. Kuwait’s economic system is heavily dependent on exploiting and commercialising oil resources, and the 2020 Global Health Crisis highlighted the urgent need for economic diversification and sector-specific risk management strategies. Policy-makers might consider assessing the country’s economic needs and identifying strategies that provide a working context towards diversification. The different responses of sectors to the dual shock presented in this paper highlight the importance of a sector-focused policy and investment strategies combined with risk management approaches that can contribute to enable a working system that can respond to exceptional challenges and opportunities assocaited with an small open economy dominated by the exports of oil. Moreover, policy-makers need to look at the regional levels in terms of opportunities and in particular, to consider approaches that contribute to the development of an active policy framework that fosters the diversification of the Kuwait economy, highlighting the sustainability challenges due to the heavy reliance on oil. For instance, Kuwait policy-makers could consider exploring the case of the UAE. The UAE is an economy rich in natural resources and has developed the second-largest economy among GCC countries, supporting a strong commitment to diversification. Its achievements in economic diversification are largely credited to a clear-cut vision and implementation of a robust legislative framework that includes regulations in various sectors of the economy, as well as policy decisions on investments and trade, simplifying the expansion of the private sector by investing in sectors such as tourism, renewable energy, finance, and technology, which have reduced its dependence on oil revenues.

**2. The first two paragraphs on page 1 correctly highlight the dominant focus of existing research on developed economies, specifically OECD countries, and identify the gap in literature concerning oil-exporting economies, particularly the Gulf Cooperation Council (GCC) economies and Kuwait. However, the background information on dual shocks, sectoral breakdown, and the linkage between the global health crisis of 2020 and its significant impact on Kuwait’s macroeconomy and stock markets is necessary to fully understand the context and importance of the study, which is not explicitly and comprehensively presented in the introduction. I understand that those information is separately presented in the subsequent two sections but the should be move here in the starting section of the introduction.**

**On page 1, the first line of paragraph 2 in the introduction mentions the dual shocks of COVID-19 and the oil price war but does not provide enough detail on how these events unfolded and their specific impacts on the global and Kuwaiti economies.**

The reviewer’s comments were addressed in the paper by adding a background on dual shocks (COVID-19 and oil price war) to the introduction, explaining their global impact and the implications for Kuwait. The outlined issues were covered by integrating and reorganising the material outlined by the reviewer as follows.

The 2020 dual shock is considered a unique global issue because, for the first time in history, the world economy faced a combination of a health crisis and an oil price crisis, which had a particular effect on Kuwait as a small oil exporting economy, disrupting socio-economic, political, and environmental dynamics. The most up-to-date research studies focusing on the analysis of the impact of the 2020 dual shocks identified as the COVID-19 pandemic and increased oil price volatility resulting from the oil price war between Saudi Arabia and Russia is dominated by studies on the world’s most developed economies represented by the OECD countries. On the other hand, oil-exporting economies have received less attention. More specifically, there is a shortage of research examining sectoral performance in the context of the GCC economies, particularly in the case of Kuwait. Furthermore, the reviewed literature revealed a dearth of research studies examining the impact of the dual market shock at market and sectoral levels.

To the best of the authors’ knowledge, this research study is the first one that examines the Kuwait stock market at a sectoral level by using a combination of well-known and established econometric methodologies to examine the impact of the dual shock, providing a unique contribution to the literature. Specifically, the current study provides empirical evidence about global factors that affect Kuwait’s stock market behaviour, which will be helpful to the country’s investors and policy-makers. The research findings can help investors regarding long-term investment planning and short-term speculation activities and help in understanding the importance of building efficiently diversified portfolios across different sectors with the aim to reduce potential market risks. Lastly, this research could also be used by governments and policy-makers when implementing future procedures and guidelines for oil-dependent countries such as Kuwait and when they design and implement strategies that enable the transition towards more sustainable economic models that can add value to countries whose economic models are heavily dependent on the export of fossil fuels. Since the discovery of oil in Kuwait in 1938, oil has played a critical role in the development of the country’s economy. According to the OPEC Annual Report (2021), Kuwait ranks among the world’s leading oil-exporting nations. In 2020, Kuwait was the world’s eighth largest producer of crude oil, valued at approximately 2,438 million dollars per day, accounting for approximately 4 percent of the world volume of crude oil exports. Moreover, Kuwait accounts for 11 percent of the OPEC member’s value of petroleum export countries, representing approximately 35,231 million US dollars, with 6 percent of the world oil reserves, by 101.5 billion barrels. The oil sector makes up nearly half of Kuwait’s GDP, around 95 percent of its exports, and approximately 91 percent of government revenue (OPEC, 2023).

Kuwait’s industrial sector accounts for 45.4 percent of its GDP and employs 25 percent of the workforce, the manufacturing sector remains underdeveloped, contributing only with 7 percent to the national GDP as reported by the (World Bank2022,2023). The year 2020 brought exceptional challenges with the outbreak of the COVID-19 pandemic and the subsequent collapse in oil prices as a result of the COVID-19 and the oil price war between Saudi Arabia and Russia that brought significant uncertainty levels to the world economies and had significant implications for oil-exporting economies as it was the case of Kuwait. The Kuwait economic model is significantly exposed to the oil market dynamics (Al-Shami and Ibrahim, 2013; Al-Hayky and Naim, 2016; Merza and Almusawi, 2016; Kisswani and Elian, 2016; Kisswani and Elian, 2017; Alshihab and Alshammary, 2020). Researchers such as Al-Yahyaei et al. (2021) and Hammami (2020) have offered evidence that Kuwait’s heavy dependence on oil-made sectors that are tied to oil and gas such as basic materials and industrials suffered significant losses resulting from the oil price collapse and the significant economic disruptions resulting from the Global Health Crisis.

During this period, the oil barrels were sold at historically low prices, as WTI recorded a historic low, entering negative territory with a -36$ price that was followed the next day with -6$ for the first time in oil history (Estrada, 2020). Hence, some sectors in the Kuwait Stock Market that are highly influenced by oil prices fell significantly; for example the consumer services, industrial and basic materials were the most affected as their prices dropped by more than 30 percent. Consumer services and industrial and basic materials sectors were identified as highly correlated to the movement of oil prices. The consumer services sector is directly exposed to oil price movements as government revenues and public spending. Consequently, consumer confidence is significantly affected by lower oil prices, which may soften the demand for consumer services. Moreover, the industrial sector is considered the most exposed sector to oil prices due to petrochemicals and refining activities that were negatively affected, as lower oil prices translate into less profits. In comparison, higher oil prices will lead to increased investment and expansion. Finally, in the case of basic materials, the cost and availability of raw materials like petrochemicals, fertilisers, and construction materials are also significantly influenced by oil prices, as many of these materials are oil derivatives (IMF, 2022, World Bank, 2023, OPEC, 2023). Similarly, Saudi Arabia was exposed to almost the same riks, variations and fluctuations as the major GCC oil exporter’s countries. Researchers such as Al-Abri and Baghestani (2021) illustrate that the Saudi Stock market sectors experienced a similar impact as Kuwait stock market sectors to the dual shock due to the economic models of both countries having a significant reliance on the dynamics and performance of the oil market.

Notably, there is a dearth of research studies examining the dynamic relationship between oil prices and Kuwaiti Stock Market sectors amidst the dual shock. This paper added value by systematically analysing how Kuwaiti stock market sectors responded to the combined shocks of the COVID-19 pandemic and the oil price war between Saudi Arabia and Russia. To the best of the authors’ knowledge, this dual shock remains underexplored in the academic literature, with a significant lack of studies on the context of Kuwait’s stock market at a sectoral level. Furthermore, there is no evidence of research that combines multiple methodologies such as the Johansen and Juselius Cointegration test (1988), Engle and Granger Causality test (1987), Granger causality test (1969), and Breitung and Candelon Frequency Domain Causality test (2006) focused on Kuwait’s stock market at a sector level.

By implementing a multiple methodologies approach, this research provides insights for investors, portfolio managers, policy-makers and academics interested in the analysis of sectoral behaviour and sensitivity of the Kuwaiti stock market in relation to oil benchmarks (Brent, Dubai, OPEC, and WTI). This study can assist investors in diversifying their portfolios and recognising the importance of hedging against market uncertainties. The combination of multiple methodologies enhances understanding of the dual shock’s effects on Kuwait’s stock market. Such insights are valuable for investors targeting oil-dependent economies like Kuwait, allowing for a multidimensional view and by offering a comprehensive insight into the impact of a dual shock in the Kuwait’s stock market. The econometric working framework enhanced the analysis’s richness while highlighting the comparative strengths of both well-established econometric models. As this research aims to empower investors with a better understanding of the details of the stock market’s response to external shocks.

**3. Like I mentioned, to address the background information consistently, I suggest moving the sections “2. Dual Shock – Insights from Global Impact towards Sectoral Level” and “Insights at the Global Markets Level” to page one after the first paragraph, incorporating the necessary information on the dual shocks and sectoral breakdown. These two sections are unnecessarily lengthy and could be significantly shortened. Additionally, the authors do not explicitly state the specific motivation for choosing Kuwait as the focus of the study. While the text mentions the global health crisis and the oil price war, it does not clearly connect these events to the specific need to study Kuwait’s stock market sectors.**

Reviewer comment addressed in the draft: Summarised and integrated key points from “Dual Shock” and “Global Markets Insights” sections into the introduction. Moreover, shortened and reorganised these sections for better flow. Furthermore, explicitly connected Kuwait’s selection to its unique challenges during the dual shocks as follows:

The 2020 global health crisis and its impact on global stock markets have attracted a significant level of attention among academics and practitioners, with a dominance of research studies focusing on the world’s most developed economies represented by the OECD countries (Alber, 2020; Vidya and Prabhesh, 2020; Morales and Andreosso-O’Callaghan, 2020; Mhalla, 2020, Alsamman and Akkas; 2022; Hung, 2020; Ozili & Arun, 2020; Minami Funakoshi, 2020; Zhang et al., 2020; Vishnoi and Mookerjee, 2020; Shehzad et al., 2020; Fernandez, 2020; Ayittey et al., 2020; Albulescu, 2021, and Al-otaibi and Morales, 2022). On the other hand, oil-exporting economies have received less attention. More specifically, there is a shortage of research examining sectoral performance in the context of the GCC economies, particularly in the case of Kuwait. Sectoral performance and sensitivity are significant areas of study as they help identify the impact of shocks on specific sectors and can contribute to developing policies that minimise potential spillover and adverse effects on the real economy. For instance, in oil-dependent countries, governments and policy-makers need to have a greater understanding of shocks in the oil sector and their spillover effects as they are essential to inform decision-making aiming to introduce economic diversity seeking to minimise potential risk exposure.

The COVID-19 global pandemic created fear and uncertainty at the socio-economic level and systemically influenced the financial system. The global health turmoil generated national spillover effects first within the context of China, where the virus originated. The health crisis grew into an economic and financial dimension of international proportions as the virus spread worldwide (Vidya and Prabhesh, 2020; Morales and Andreosso-O’Callaghan, 2020; Al-otaibi and Morales, 2022). Therefore, some restrictions on economic activities were required to control the spreading of COVID-19, and these actions negatively affected the global demand for leisure, tourism and travel sectors. The market dynamics led to a sharp decrease in consumption and economic production for all businesses and industries, significantly affecting global supply chains (Mazur, Dang & Vega, 2020; Malla, 2020). Hence, this decrease also negatively affected the dynamics of the economic and financial sectors (Zhang, Hu, & Ji, 2020; Ayittey et al., 2020). Furthermore, a drop in oil prices followed on March 9, 2020, due to a fall in global demand triggered by the oil price war between Saudi Arabia and Russia, created further pressures in countries’ economic systems. As a result, there was a decline in oil prices by more than 20 percent in one day (Jawadi and Sellami, 2021). Hence, in April 2020, the West Texas Intermediate (WTI) oil prices became negative for the first time in history due to declining demand (BBC, 2021; Al-otaibi and Morales, 2022). This can be attributed to the economic slowdown that occurred because of the impact of COVID-19 (Gharib, Mefteh-Wali, & Jabeur, 2020). The global financial market was negatively impacted due to the reduction of oil demand in the commodity markets, especially in oil prices, as a result of the restrictions introduced to try and curtail the spread and impact of the pandemic. This unexpected decline reflects a significant sharp decline as the prices and stock market positions faced a dual challenge to policy-makers trying to stabilise the markets as they are mutually dependent (Hung, 2020). From this outlook, it should be mentioned that the impact of oil prices on the stock market due to the impact of COVID-19 on the economy has a significant impact on oil-exporting countries. Thus, the stock markets, particularly in oil-dependent countries, have been strongly negatively impacted by the dual shock that the world experienced early in 2020 (Prabheesh, Garg & Padhan, 2020b; Albulescu, 2020; Huang, & Zheng, 2020; Alsamman et al., 2022). Among these oil-dependent countries is Kuwait, which registered a significant drop in its All-share index by 25 percent on March 23, 2020 (Al-otaibi and Morales, 2022).

**4. I suggest that the authors provide a more detailed elaboration on Kuwait’s economic structure, its dependence on oil, and the specific vulnerabilities it faced during the dual shocks. While the introduction acknowledges a scarcity of research on sectoral performance in GCC economies, it lacks specificity regarding the findings of existing studies and how this study intends to address this gap.**

The reviewer comment was addressed in question 2 as follow:Since the discovery of oil in Kuwait in 1938, oil has played a critical role in the development of the country’s economy. According to the OPEC Annual Report (2021), Kuwait ranks among the world’s leading oil-exporting nations. In 2020, Kuwait was the world’s eighth largest producer of crude oil, valued at approximately 2,438 million dollars per day, accounting for approximately 4 percent of the world volume of crude oil exports. Moreover, Kuwait accounts for 11 percent of the OPEC member’s value of petroleum export countries, representing approximately 35,231 million US dollars, with 6 percent of the world oil reserves, by 101.5 billion barrels. The oil sector makes up nearly half of Kuwait’s GDP, around 95 percent of its exports, and approximately 91 percent of government revenue (OPEC, 2023).

Kuwait’s industrial sector accounts for 45.4 percent of its GDP and employs 25 percent of the workforce, the manufacturing sector remains underdeveloped, contributing only with 7 percent to the national GDP as reported by the World Bank2022,2023. The year 2020 brought exceptional challenges with the outbreak of the COVID-19 pandemic and the subsequent collapse in oil prices as a result of the COVID-19 and the oil price war between Saudi Arabia and Russia that brought significant uncertainty levels to the world economies and had significant implications for oil-exporting economies as it was the case of Kuwait. The Kuwait economic model is significantly exposed to the oil market dynamics (Al-Shami and Ibrahim, 2013; Al-Hayky and Naim, 2016; Merza and Almusawi, 2016; Kisswani and Elian, 2016; Kisswani and Elian, 2017; Alshihab and Alshammary, 2020). Researchers such as Al-Yahyaei et al. (2021) and Hammami (2020) have offered evidence that Kuwait’s heavy dependence on oil-made sectors that are tied to oil and gas such as basic materials and industrials suffered significant losses resulting from the oil price collapse and the significant economic disruptions resulting from the Global Health Crisis.

During this period, the oil barrels were sold at historically low prices, as WTI recorded a historic low, entering negative territory with a -36$ price that was followed the next day with -6$ for the first time in oil history (Estrada, 2020). Hence, some sectors in the Kuwait Stock Market that are highly influenced by oil prices fell significantly; for example the consumer services, industrial and basic materials were the most affected as their prices dropped by more than 30 percent. Consumer services and industrial and basic materials sectors were identified as highly correlated to the movement of oil prices. The consumer services sector is directly exposed to oil price movements as government revenues and public spending. Consequently, consumer confidence is significantly affected by lower oil prices, which may soften the demand for consumer services. Moreover, the industrial sector is considered the most exposed sector to oil prices due to petrochemicals and refining activities that were negatively affected, as lower oil prices translate into less profits. In comparison, higher oil prices will lead to increased investment and expansion. Finally, in the case of basic materials, the cost and availability of raw materials like petrochemicals, fertilisers, and construction materials are also significantly influenced by oil prices, as many of these materials are oil derivatives (IMF, 2022, World Bank, 2023, OPEC, 2023). Similarly, Saudi Arabia was exposed to almost the same riks, variations and fluctuations as the major GCC oil exporter’s countries. Researchers such as Al-Abri and Baghestani (2021) illustrate that the Saudi Stock market sectors experienced a similar impact as Kuwait stock market sectors to the dual shock due to the economic models of both countries having a significant reliance on the dynamics and performance of the oil market.

Notably, there is a dearth of research studies examining the dynamic relationship between oil prices and Kuwaiti Stock Market sectors amidst the dual shock. This paper added value by systematically analysing how Kuwaiti stock market sectors responded to the combined shocks of the COVID-19 pandemic and the oil price war between Saudi Arabia and Russia. To the best of the authors’ knowledge, this dual shock remains underexplored in the academic literature, with a significant lack of studies on the context of Kuwait’s stock market at a sectoral level. Furthermore, there is no evidence of research that combines multiple methodologies such as the Johansen and Juselius Cointegration test (1988), Engle and Granger Causality test (1987), Granger causality test (1969), and Breitung and Candelon Frequency Domain Causality test (2006) focused on Kuwait’s stock market at a sector level.

By implementing a multiple methodologies approach, this research provides insights for investors, portfolio managers, policy-makers and academics interested in the analysis of sectoral behaviour and sensitivity of the Kuwaiti stock market in relation to oil benchmarks (Brent, Dubai, OPEC, and WTI). This study can assist investors in diversifying their portfolios and recognising the importance of hedging against market uncertainties. The combination of multiple methodologies enhances understanding of the dual shock’s effects on Kuwait’s stock market. Such insights are valuable for investors targeting oil-dependent economies like Kuwait, allowing for a multidimensional view and by offering a comprehensive insight into the impact of a dual shock in the Kuwait’s stock market. The econometric working framework enhanced the analysis’s richness while highlighting the comparative strengths of both well-established econometric models. As this research aims to empower investors with a better understanding of the details of the stock market’s response to external shocks.

**5. It is probably better to introduce the key findings early in the introduction, followed by their implications for potential policy recommendations arising from the study. The discussion should emphasise how understanding sectoral sensitivity to shocks can inform policies aimed at economic diversification and risk mitigation in oil-dependent economies. Furthermore, the study should outline its contributions to the existing literature on the impact of dual shocks on stock markets, highlighting new insights into the sectoral performance of Kuwait’s stock market.**

The research findings offer evidence of a significant impact of the four selected oil benchmarks (Brent, Dubai, OPEC and WTI) on the behaviour of Kuwait’s stock market sectors, highlighting the country’s heavy dependence on its oil and signalling the rising need for economic diversification and sustainable development. Sectoral performance needs to be carefully considered in terms of policies that minimise the impact of oil fluctuations and the spillover effects resulting from diverse shocks and crises and their implications on sectors. This research study offers insights into how basic materials and industrials emerged as heavily exposed to oil price fluctuations due to the sector’s heavy reliance on raw materials and energy costs, signalling the importance of active policies that could lead to interventions seeking to minimise losses. Kuwait policy-makers need to consider the lack of diversification in Kuwait’s economy and uplift the non-oil sectors to increase economic growth and ensure that Kuwait’s exposure to the oil sector and associated risks is addressed within the context of the global sustainability goals.

The methodological framework used in this paper offers a critical assessment of selected econometric models that can help better understand the impact of the dual market shock that took place in 2020, with the outburst of the Global Health Crisis, namely, COVID-19, as well as the oil price war between Russia and Saudi Arabia and its implications for the Kuwait stock market. The reviewed acadamic literature confirmed that cointegration and causality models’ are highly effective in analysing the static and dynamic relationship between oil prices and the Kuwaiti Stock Market. Al-Shami and Ibrahim’s (2013) report a positive relationship between oil prices, money supply, inflation, and stock returns in Kuwait exhibiting bi-directional causality between the variables from 2001 to 2010. Moreover, Al-Hayky and Naim’s (2016) identified an existing long-term positive relationship between oil price and the stock market of Kuwait during high volatility periods and a non-existent one during periods of low volatility (2006–2015). On the other hand, both Merza and Almusawi (2016) and Kisswani and Elian (2017) analysed the relationship between oil prices and the Kuwait stock market on a sectoral base. Their research findings revealed that oil prices as a macroeconomic factor drive Kuwait’s banking, real estate, and insurance sectors. Moreover, Kisswani & Elian (2018) found evidence of a long-run relationship between oil prices-Brent and WTI-and Kuwaiti stock market returns, where daily shocks in oil prices negatively affect the returns. Their sectoral analysis for 2000-2015 showed asymmetric long-run effects between oil prices and some Kuwaiti sectors, while asymmetry in the short run was observed only for WTI but not for Brent. Alkandari & Abul (2019) confirm a short-run relationship between oil and Kuwait stock market during the period 2005 to 2018. The analysis of Alshihab and Alshammari (2020) offered evidence of the fluctuations of the oil prices on the Kuwait stock market returns for the period of 2000 to 2020. In the long run, the findings confirmed that changes in Kuwaiti stock market returns are only affected by oil price fluctuations in the short run. Moreover, Algahtani et al. (2019) examined the impact of volatility in oil prices on GCC sectoral returns using frequency domain causality. The results depicted interdependence across most sectors except energy and transportation. Moreover, Almassari et al. (2020) used the Frequency Causality Domain model to test the relationship between economic growth and financial developments in Kuwait. Therefore, the reviewed literature illustrates a dearth of research studies examining volatility patterns in the context of the Kuwaiti Stock Market and the differences between static and dynamic relationships between oil prices and Kuwaiti Stock Market Sectors amidst the dual shock. Moreover, to the best of the author’s knowledge, it is the first time that this test is used in combination with Johansen Cointegration test (1988), Engle and Granger (1987), Granger causality test (1969) and the frequency domain causality test, Breitung and Candelon (2006) methods in the context of Kuwait stock market at a sectoral level. Therefore, this study examines the Kuwait stock market at a sectoral level during the COVID-19 pandemic and the oil price war simultaneously in 2020, contributing to filling the identified research gap. Moreover, from a methodological viewpoint, combining several models ensures the robustness and rigour of the econometric framework and allows for a more nuanced understanding of sectoral sensitivities. This multi-benchmark approach provides policy-makers with reliable data to design risk mitigation strategies. The study findings have flagged the importance of policies aimed at economic diversification and sustainable development in line with global sustainability goals and the importance of integrating diverse modelling processes that help to cross-check research outcomes.

**6. Prior to examining into the data and methodology section, I suggest the authors develop a section outlining their research questions and potential hypotheses for exploration in this paper. The hypothesis section is currently absent.**

The research question under consederation:

1. **What is the impact of the 2020 Dual market shock (COVID-19 and oil prices shock) on Kuwait stock market sectors?**

H10: There is evidence of a potential long run relationship between oil benchmarks and Kuwait stock market sectors.

H1A: There is no evidence of a potential long run relationship between oil benchmarks and Kuwait stock market sectors.

H20: There is evidence of a potential short run relationship between oil benchmarks and Kuwait stock market sectors.

H1A: There is no evidence of a potential short run relationship between oil benchmarks and Kuwait stock market sectors.

The main objective of this paper is to understand the impact of oil prices on Kuwait stock market sectors during the dual market shock (COVID-19 and oil prices shock) and to provide insights that add value to investment portfolio managers, long and short term investors and policy-makers regarding Kuwait’s stock market and sectors behaviour. Understanding the dynamics and the response of the stock market indices/sectors during periods of high volatility can help with decision-making and portfolio selection, along with long and short-term investment plans that integrate rich oil-exporting economies.

**7. I suggest that the authors briefly provide the rationale behind their decision to begin the analysis from December 31, 2015, focusing on the Kuwait Weighted Market Index, sectoral indices, and crude oil benchmarks. It is unclear why the authors omitted data preceding 2015, especially when such observations could readily be retrieved from DataStream.**

**They should discuss any significant economic events or market conditions around that time that justify this starting point. Additionally, they should explicitly connect the selection of these specific indices and benchmarks to the research objectives, explaining how these choices will facilitate understanding of sectoral sensitivity to the dual shocks of COVID-19 and the oil price war. The section should also include a key summary statistics table, providing essential insights into the summary statistics.**

The data set consists of the daily closing price, representing a total of 1,604 observations for the Kuwait stock market All Share Index and all the Kuwait stock market sectors. The reviewed literature suggests that the required sample size to implement cointegration and causality models requires a minimum of 100 observations(Aguiar-Conraria & Soares, 2014; Percival & Walden, 2020). However, researchers recommed working with larger samples as they are considered to be more reliable (Toda & Yamamoto, 1995; Percival & Walden, 2000; Lütkepohl, 2005; Nielsen, 2010; Hacker & Hatemi-J, 2012; ; Toda & Phillips, 2014; Asteriou & Hall, 2015; Gómez & Maravall, 2015; Gredenhoff & Karlsson, 2018; ). Additionally, the time period under consideration spans from December 31 2015 to February 23, 2022. The analysis starts from December 31, 2015, to avoid introducing undesired noise into the sample resulting from the 2014 oil prices shocks and the lagging effects of the global economic recovery from the 2007/08 Global Economic and Financial Crisis. These shocks resulted in extreme volatility in global oil markets and profoundly impacted oil-dependent economies such as Kuwait, as noted by Masood et al. (2019); Cause (2015); Fattouh, (2016). Including the data before 2015 could lead to misrepresentation of the issues under consideration by conflating lagged effects of earlier shocks with the 2020 dual shock, (COVID-19 and the oil price war) under examination. The selection of the Kuwait weighted market index, sectoral indices and crude oil benchmarks (Brent, Dubai, OPEC and WTI) aligns with the study objective of analysing the reaction of the stock market indices/sectors during the dual shock. Additionally, stopping the time frame to February 23, 2022, to avoid the influence of the Russian-Ukrainian war on oil prices that started on February 24, 2022 (Zhang, et al., 2024; Gaidzik et al., 2024).

**8. On page 7, the authors mention consideration of three well-known stationarity tests: the Augmented Dickey-Fuller (ADF) test (1979), Phillips-Perron (PP) test (1988), and Kwiatkowski-Phillips-Schmidt-Shin (KPSS) test (1992). I suggest they present the key insights of the stationarity tests in this section, with detailed tables related to these tests placed in the appendix instead of the table currently located on page 18.**

Reviewer comment addressed in the draft: Summarised insights from stationarity tests (ADF, PP, KPSS) in the discussion on page 7 and moved detailed tables to the appendix for clarity. The tests show that prices series for Kuwait sectors, Oil benchmarks are non-stationary in levels, but are stationary at 1% level in returns. Moreover, the p-values results shown in parentheses; there is no p-value for KPSS; therefore, the 1% significance level was considered for the test at a value of 0.739000. The main indices for the sectors and major oil Benchmarks are presented, and the results for the three stationarity tests with p-values in brackets and the number of required lags to estimate the tests are reported in the table.

**9. The analysis of the three subsections “3.1.1 Cointegration,” “3.1.2 Causality,” and “3.1.3 Frequency Domain Model” should be more closely aligned with the research questions and hypotheses of the authors. I suggest they provide a comprehensive discussion of these sections in relation to their research hypotheses, and consider condensing them for clarity.**

To address the reviewer comment we have restructured and refined the analysis of subsections “3.1.1 Cointegration,” “3.1.2 Causality,” and “3.1.3 Frequency Domain Model”. This revision aligned with the research questions and hypotheses.

To answer the research main question:

**What is the impact of the 2020 Dual market shock (COVID-19 and oil prices shock) on Kuwait stock market sectors?**

The hypothesis under consideration in this case are as follows:

H10: There is no evidence of a potential long run relationship between oil benchmarks and Kuwait stock market sectors.

H1A: There is no evidence of a potential long run relationship between oil benchmarks and Kuwait stock market sectors.

H20: There is no evidence of a potential short run relationship between oil benchmarks and Kuwait stock market sectors.

H1A: There is no evidence of a potential short run relationship between oil benchmarks and Kuwait stock market sectors.

Each hypothesis is now addressed separately with supporting evidence and explanation derived from the analysis in subsection 3.1.1, 3.1.2 and 3.1.3.

**Subsection analysis**

**3.1.1 Cointegration**

The Cointegration test investigates the long-run relationship between oil benchmarks and Kuwait’s stock market sectors. The results illustrate whether a statistically significant long-run relation exists by testing the outlined null and alternative hypotheses.

- If Cointegration is detected, H10 is supported and indicates that oil benchmarks and Kuwait stock market sectors share a stable long-run connection.
- If no Cointegration is found, H1A is accepted, rejection of long-run connection between oil benchmarks and Kuwait stock market sectors.

We provide a detailed interpretation of the test statistics Engel-Granger and Johansen tests and their implications for long run relationships, explicitly trying these to research hypothesis as follows:

Cointegration test is used to investigate the presence of a long-term relationship between variables. The Cointegration analysis examined the association between oil benchmarks (Brent, Dubai, OPEC and WTI) and Kuwait stock market sectors. The Kuwait All-share Index illustrates some interesting insights, as Kuwait’s stock market sectors had different long-term relationships with the oil benchmarks included in this study. Hence, the cointegration test (see Appendix 2 Table 3 in the manuscrip) identifies that WTI oil benchmark is cointegrated with ten out of twelve Kuwait stock market sectors, including the All-share index (Industrial, Basic Materials, All share index, Banks, Financial Services, Real Estate, Oil & Gas, Healthcare, Telecommunications, Insurance and Technology). Moreover, Brent oil benchmark is cointegrated with eight sectors (Industrial, Basic Materials, Healthcare, Real Estate, Oil & Gas, Telecommunications, Insurance and Technology). The OPEC oil benchmark is cointegrated with five sectors (Basic Materials, Healthcare, Oil & Gas, Telecommunications, and Technology). The Dubai oil benchmark is cointegrated with seven sectors (Industrial, Healthcare, Basic Materials, Oil & Gas, Telecommunications, Insurance, and Technology). Hence, the Cointegration test (table 7 in the draft) shows that Telecommunications, Oil & Gas, Healthcare, Basic Materials and Technology are cointegrated with all oil benchmarks.

**3.1.2 Causality**

The Causality analysis investigates the short run relationship between oil benchmarks and the Kuwait stock market sectors. The results illustrate whether a statistically significant short-run relation exists, by addressing H20 and H2A:

- If Causality is detected, H20 is supported, indicating that oil benchmarks and Kuwait stock market sectors share a short-run connection.
- The absence of Causality supports H2A rejection of the short-run connection between oil benchmarks and Kuwait stock market sectors.

We provide a detailed interpretation of the test Granger causality test (1969) and its implications for short run relationships, explicitly trying these to research hypothesis as follows:

The Causality test is used to investigate the presence of a short-term relationship between variables. The causal analysis between oil benchmarks (Brent, Dubai, OPEC and WTI) and Kuwait stock market sectors, including the Kuwait All-share index, offer significant evidence suggesting that the Kuwait stock market sectors exhibited a different short-term relationship towards different oil benchmarks. Hence, the causality test (Appendix 2 Table 4 in the draft) identifies that the WTI oil benchmark is causal with five sectors (Basic Materials, Financial Services, Real Estate, Telecommunications, and Technology). The Brent oil benchmark is causal with six sectors (Industrial, Basic Materials, All share index, Banks, Financial Services, Technology). In addition, the OPEC oil benchmark is causal with seven sectors (Industrial, Basic Materials, All Share Index, Banks, Financial Services, Real Estate, Oil & Gas). The Dubai oil benchmark is causal with seven sectors (Consumer Services, Industrial, Basic Materials, All share index, Banks, Financial Services, Real Estate, and Consumer Goods). The causality test illustrates that Basic Materials, Financial Services and the Real Estate sectors are causal with the four oil benchmarks, illustrating a statistically significant short-run relationship.

**3.1.3 Frequency Domain Model**

The frequency domain causality test was applied to understand the dynamic short-run relationship between oil benchmarks in Kuwait’s stock market sectors. It complement the findings in subsection 3.1.2 Causality test.

The frequency domain causality test results are linked back to the research questions and hypotheses, illustrating how these findings refine our understanding of short-run relationship between oil benchmarks in Kuwait’s stock market sectors, as our results supported causality findings, illustrating no causality between oil benchmarks and the insurance sector and technology. Furthermore, the results from the domain causality modelling illustrate causality between oil benchmarks and real estate and financial services sectors. Interestingly, the research findings provide evidence of the strong correlation between oil benchmarks and the basic materials index and a long-term and short-term relationship between oil benchmarks and the basic materials index. However, from a macroeconomic prospective basic materials sector is not significant to Kuwait stockmarket as it has ‘small weight’ as market capitalization that is dominated by banking sector (Boursa Kuwait –Annual Report 2020,2021,2022)

**10. The methodology employed includes traditional Cointegration and Causality models, alongside the dynamic causal modeling of short-run dynamics introduced by Breitung and Candelon (2006). However, the section lacks mention of any assumptions or limitations associated with these models, including the frequency domain model. Addressing these would enhance transparency in the research methodology.**

**As a reader, I am more interested to understand how the use of Cointegration and Causality models, as well as the Breitung and Candelon’s frequency domain model, represents a novel approach to data analysis compared to more advanced methodologies currently employed in similar research.**

Concerning the reviewer comment by providing the assumptions, limitations, and comparative novelty of the models we have employed.

Assumptions and Limitations

The methodologies used in this study are based on well-established econometric models, each with its own set of assumptions and limitations. Here are the details:

- Cointegration Models:

Assumptions: Cointegration analysis assumes that data series are non-stationary and integrated of the same order, usually integrated processes of order one or I(1) processes (Engle & Granger, 1987). The Johansen test requires large sample sizes for reliable results and assumes no structural breaks in the data (Johansen, 1991).

Limitations: Small sample sizes can affect the power of the test, potentially leading to misidentification of Cointegration relationships (Lütkepohl, 2005).

- Causality Models:

Assumptions: Granger causality tests assume linear relationships and stationarity of the time series data (Granger, 1969). Proper lag length selection is crucial as it significantly influences results (Lütkepohl, 1991). In the research context of the paper, the VAR modelling approach was considered to ensure the identification of optimal lags to be considered in cointegration and causality models.

Limitations: These tests predict rather than establish true causality and may fail to detect non-linear dependencies (Toda & Yamamoto, 1995). Moreover, they might overlook dynamic, time-varying relationships in high-frequency data (Balcilar et al., 2010).

- Breitung and Candelon’s Frequency Domain Model:

Assumptions: This model relies on the stationarity of the series and proper lag structure specification. It also assumes that causal relationships vary by frequency instead of being constant over time (Breitung & Candelon, 2006).

Limitations: Frequency domain models can be sensitive to model specification errors, complicating interpretation (Wang et al., 2020). Moreover, the methodology might not account for non-cyclic relationships or those outside the specified frequency ranges (Fan & Yao, 2003).

Comparative Analysis

The innovative aspect of using Cointegration, Causality, and the Breitung and Candelon frequency domain model together lies in the Integration of Long- and Short-Run Dynamics, as Traditional studies often focus on either long-term equilibrium relationships (e.g., Cointegration) or short-term interactions (e.g., Granger Causality). By employing both econometric modelling processes, this study bridges the gap between these static long and short-run relationships and integrates a additional dimension that accounts for dynamic short-run interactions, providing a more comprehensive understanding (Engle & Granger, 1987; Toda & Yamamoto, 1995). Moreover, the integration of the Frequency Domain modelling add additional insights by adding a causal dynamic context as introduced by Breitung and Candelon (2006) in their frequency domain model that captures causal relationships across different time frequencies, which is particularly relevant for financial markets where relationships between variables often change over time (Balcilar et al., 2010). This approach offers deeper insights into cyclical behaviours and how oil price changes affect stock market sectors differently over various time horizons (Wang et al., 2020).

Justification for Model Selection

The methodological choices align with our research objectives as Traditional Econometric Models provide a solid foundation for analysing both long- and short-term relationships. Moreover, the Frequency Domain Approach helped in extending traditional models, allowing us to capture temporal variations in causal relationships within a dynamic context. Hence, by combining these methodologies, we address both static and dynamic relationships, ensuring methodological rigour and relevance to our research questions.

**11. Figure 1 should not serve as the starting point for the “Core Research Findings.” Instead, I suggest integrating this figure into the data section, where the key summary statistics table is currently absent. Similarly, Figure 2 and its descriptive content should be relocated to the data section, with Figure 2 ideally placed in the appendix.**

Reviewer comment addressed in the draft: Relocated Figure 1 to the data section and, added a summary statistics table and moved Figure 2 to the appendix to streamline the core findings section.

**12. Pages 19 to 20 present the key results of the Cointegration & Causality Findings. However, the explanations provided in this section are purely of a statistical nature and lack a connection to the research questions and hypotheses. It is essential to justify these findings from the perspective of economic and financial theories, which is currently absent. Also, I find this section is bit incomplete.**

Cointegration is a statistical concept used to describe the long-term relationship among time series variables that exhibit trends, either deterministic or stochastic. The research paper is supported by the Engle-Granger method to explore whether a long-run association exists between the variables, and the Johansen cointegration test was applied for comparison purposes. Several notable studies, including those by Granger, Huangb, and Yang (2000), Miller and Ratti (2009) and Muhtaseb and Al-Assaf (2016), have used these approaches to examine long-term relationships between oil prices and stock markets across various economies and markets including Kuwait. These studies provide contemporary evidence of the value and significance of these econometric models.

Relating to Economic and Financial Theories, the Cointegration tests’ results suggest a long-run equilibrium relationship between oil prices and stock markets. This relationship has corresponding implications with Frank Knight’s Risk and Uncertainty Theory (1924) in which measurable risk is separated from unmeasurable uncertainty. The cointegration relationship indicates that although a few trends or patterns in oil prices could be measured and expected (risk),  an unpredictable component (uncertainty) that affects the stock market sectors exists. Granger causality testing was also applied to address linear prediction, particularly in cases where one event precedes another. It is often observed that unknown external factors influence variables. Research by Huang, Masulis, and Stoll (1996) and Lee, Yang, and Huang (2012) used Granger causality to investigate the relationship between oil and stock returns. Additionally, frequency domain causality was utilised to analyse the dynamic interactions between stock returns and Brent oil prices. Many studies have employed the outlined techniques and reported significant findings (Gradojevic, 2013; Ozer and Kamisli, 2016).

This paper examines the long-run and short-run relationship between oil prices and stock markets, allowing investors to identify the risk and uncertainty components that drive market dynamics. This distinction is of fundamental importance to investors who must comply with decision-making pressures under the influence of both measurable risk and unmeasurable uncertainty.

**13. The paper lacks robustness analysis, making it impossible to assess the validity of the results. It remains unclear how sensitive these findings are to changes in data points or subsections of the data. Furthermore, the portfolio and investment decision implications of these findings are not discussed. Without clear portfolio implications stemming from robust findings, the authors should refrain from claiming scientific significance in contemporary literature.**

The paper acknowledges the critical importance of robustness in time series analysis to ensure the stability and accuracy of results. Therefore, by implementing a combination of econometric models between Johansen’s (1988), Engel-Granger’s (1987) Cointegration and Granger causality tests (Granger, 1969), and the frequency Causality Domain model by Breitung and Candelon (2006) provides a comprehensive framework for understanding both long-run equilibrium and short-run interactions. This multiple methods ensure reliable and stable results. This approach contributes to academic literature such as the studies developed by Algahtani et al. (2019) Abdulrazzaq et al. (2018) and Alshammari et al. (2020).

The Kuwait stock market sectors can be viewed as an attractive destination for investors when they develop their portfolios because the Kuwait Stock Market Sectors have different types of behaviour when compared to each other and the Kuwait Stock Market All-share index. Therefore, understanding the long-term and short-term relationship between oil benchmarks and Kuwait’s stock market sector indices can greatly benefit portfolio management and decision-making practices by enriching the decision-making process and supporting investors to capture potential losses and investment opportunities, monitoring their investments, and considering the importance of hedging techniques and strategies to counter and manage the effect of market uncertainty. Furthermore, it supports strategic planning and helps to understand the behaviour and performance of oil prices and oil-dependent industries, investors, speculators and other oil market participants. In turbulent times, uncertainty and business risk grow, and investors must pay more attention to business tactics and hedging. Good decision-making can vastly reduce risk and lead to mitigation of strategies that materialise significant losses. Therefore, this research paper’s outcomes can contribute to a better understanding of the impact of oil prices on the Kuwait stock market sectors and prevent the damage and high costs associated with business environments affected by risk and uncertainty derived from oil shocks.
