# Supplementary material for: Sectoral sensitivity of the Kuwait stock market to a dual shock
Source: PLoS One. 2025 Sep 24;20(9):e0331384. doi: 10.1371/journal.pone.0331384 (PMC12459840; doi:10.1371/journal.pone.0331384)
Supplement: Attachment — Submitted filename: response to Referee report_Talal.docx [file pone.0331384.s020.docx]

Public Library of science (PLOS)

Subject: Original manuscript resubmission

Dear Plos One Editors,

We would like to thank you for all the comments and feedback on our manuscript. We have completed all requested changes and have developed a comprehensive report to support our manuscript resubmission.

The core points to consider include:

1. Updated manuscript with track changes to ensure that you are able to identify all amendments and additional information.
2. Updated manuscript without track changes.
3. Response letter with all comments addressed and full explanations provided that you can find below.

We remain at your disposal for any further comments or clarifications that might be needed.

Yours sincerely,

Talal Alotaibi

**Referee report**

**PONE-D-23-38848R1**

**Sectoral Sensitivity of the Kuwait Stock Market to a Dual Shock**

**I acknowledge the authors’ efforts in addressing most of my previous comments. However, the revised manuscript has introduced new issues regarding organization, redundancy, overly lengthy and mechanical explanations, and repetitive content across several sections, making it difficult to identify key information. For instance, the abstract could be more concise without losing essential details. A clearer opening sentence might be: “*This study examines Kuwait’s stock market sectors’ response to the dual shock, employing traditional cointegration and causality models alongside dynamic causal modeling to analyze short-run dynamics through frequency domain analysis.*” Beyond this, I find only four sentences are necessary (and sufficient) for the abstract.**

**Below are additional points that may help improve the manuscript:**

1. **Overall, the manuscript is not yet at the level expected for a scientific journal publication. The abstract and introduction are particularly critical, yet I find it challenging to distinctly and consistently convey the following:**

- **What the research question is (looks like this appears acceptable now) and the related hypotheses to be tested.**

Response: The research question:

What is the impact of the 2020 Dual market shock (COVID-19 and oil prices shock) on Kuwait stock market sectors?

Hypotheses:

1. H10: There is evidence of a potential long-run relationship between oil benchmarks and Kuwait’s stock market sectors.

H1A: There is no evidence of a potential long-run relationship between oil benchmarks and Kuwait’s stock market sectors.

2. H20: There is evidence of a potential short-run relationship between oil benchmarks and Kuwait’s stock market sectors.

H1A: There is no evidence of a potential short-run relationship between oil benchmarks and Kuwait’s stock market sectors.

- **Its significance and why it’s important (considering Kuwait, the Middle Eastern economy, and potentially the global economy).**

**Response:**

We thank the referee for this suggestion. This paper has significant implications for oil-dependent economies in the GCC and Middle Eastern regions as they are dominant players in oil production and holding reserves. The implications at the global level are paramount due to the global economy’s significant dependency on oil (Statista, 2024; OPEC 2023, World Bank, 2023; IMF, 2022). The dual shock of the COVID-19 pandemic and oil price war exposed critical vulnerabilities in mono-economies, revealing the urgent need for economic diversification and sectoral resilience (OPEC, 2023; World Bank, 2022). Kuwait’s experience offers insights into Middle Eastern economies and the GCC countries facing similar oil dependencies (Alshihab & Alshammari, 2020).

- **The methodology and rationale (how and why the chosen approach is appropriate and why did the author choose very basic time series approaches instead of frequently used advanced techniques, why do the robustness tests are not necessary for the benchmark results).**

**Response:** We thank the referee for this suggestion. The paper acknowledges the critical importance of robustness in time series analysis to ensure the stability and accuracy of results. Therefore, the methodology follows a standard econometric process: we first establish stationarity properties using the (ADF/PP/KPSS) tests, which are the most well-known and popular strategies for testing the stationarity of time series (Enders, 1995; Al khazali, 2006; Asterious and Hall, 2007, 2011; Taheri, 2014). These tests are used to examine whether there is a unit root stationarity in the chosen daily market index return and oil returns. Next, we use information criteria to choose model lags, as lag length selection is data driven and follows best practice to ensure each model is correctly specified before interpretation. We then applied a combination of econometric models, including Johansen’s (1988), Engel-Granger’s (1987) Cointegration and Granger causality tests (Granger, 1969), and the frequency Causality Domain model by Breitung and Candelon (2006). The methodological framework provides a comprehensive approach for understanding both long-run equilibrium and short-run interactions to examine the dual shock’s impact on Kuwait’s stock market sectors. These techniques were selected for their proven efficacy in capturing short-long relationships and time-based dynamics in financial markets, particularly in small, oil-exporting economies.

Our approach aligns with previous research in the region (e.g., Alshihab & Alshammari, 2020; Kisswani & Elian, 2017), ensuring methodological consistency and facilitating meaningful comparisons. Although more advanced techniques, such as machine learning, the ARDL by Pesaran (1999, 2002) and Pesaran, Shin, and Smith (2001), or more innovative models integrating novel approaches, we chose classical Cointegration and causality approaches due to their efficiency and strong theoretical foundations. This was needed to provide clear insights to policy-makers and investors, in contexts where oil leads economic activity, simpler, clear models and provide results that are more accurate without introducing unnecessary complexity (Asteriou & Hall, 2011; Toda & Yamamoto, 1995). Future research can indeed consider the development of comparative analysis between different econometric models, but this was not the purpose of this study.

The methodological framework is robust and consistent with the literature as different models were implemented to ensure robustness, consistency and validity of the results. Traditional robustness checks that include testing the residuals for serial correction, and heterokedascitity do not apply to the selected model as they are implemented using the variables in levels and the outcomes of standard robustness checks will lead to invalid results as well documented in the literature. A number of empirical studies across the financial and economics literature have employed time series methodologies such as Cointegration techniques, Granger causality tests, and unit root test using a combination of models to address the outlined issues regarding residuals checks, that are addressed by implementing different models that enable ensuring consistency of results. These studies often rely on the theoretical reliability and practical effectiveness of classical models, particularly the Engle-Granger and Johansen Cointegration frameworks, particularly in examining the long-run and short-run relations between oil prices and stock market performance. For instance, Alshihab and AlShammari (2020) applied Johansen Cointegration and Granger causality methods, supported by ADF and PP tests, to explore oil–stock relationships in Kuwait without reporting supplementary robustness tests for residuals as they are not valid for the selected models. Similarly, Kisswani and Elian (2017) adopted Johansen Cointegration and Granger causality in a nonlinear ARDL framework, and did not integrate residuals checks. Prabheesh et al. (2020) used ADF and PP tests along with Johansen’s method but also refrained from further validation. These decisions are consistent with a pattern observed in many oil-market studies that acknowledge the limitations of some time series models regarding robustness checks that are addressed with the implementation of a variety of econometric models to ensure consistency and robustness while prioritising clarity and model interpretability, particularly when the research context involves data constraints or focused sectoral analysis. Alsamman and Akkas (2022) followed a similar path, using Engle-Granger Cointegration and Granger causality models to study the economic impact of COVID-19 and oil shocks on GCC countries, without robustness extensions due to the outlined limitations. In broader global contexts, Huang et al. (1996) and Jimenez-Rodriguez and Sanchez (2005) also employed Granger causality and Cointegration analyses without incorporating parameter stability or structural break tests, reflecting a reliance on classical inference standards. Additional studies support this trend. Like, Arouri et al. (2011) examined oil stock return and volatility spillovers in GCC economies using Johansen and Granger frameworks without reporting diagnostic checks for VAR stability, Maghyereh et al. (2017) focused on volatility spillovers using Johansen Cointegration and Causality, again without validating spillover dynamics with alternate specifications. Moreover, Trabelsi (2017) and Sim and Zhou (2015) also relied on cointegration and quantile-specific Granger causality approaches, respectively, without conducting further robustness tests. Beyond the Gulf region, initial work by Imarhiagbe (2010), Serletis and Banack (1990), Quan (1992), and Schwartz and Szakmary (1994) applied similar methods in analysing petroleum markets and futures linkages without robustness testing. Furthermore, Dwipraptono (2007), Fernando and Sharpe (2015), Tran and Dao (2023), and Dai et al. (2014), which apply cointegration and causality models without using robustness checks, especially when results across core tests were consistent, as researchers acknowledge the complexities of time series analysis that in the context of the used models requires a different approach than the “traditional” tests for robustness. The papers integrates a multimodelling research framework to ensure that shortcoming regarding robustness and diagnostics checks are addressed.

Finally, these examples illustrates that the absence of robustness checks does not indicate methodological weakness. By combining theoretically justified econometric models, our study delivers reliable findings. It offers a clear analysis of the impact of the 2020 Dual market shock (COVID-19 and oil prices shock) on Kuwait stock market sectors.

- **Key findings, novelty, and contributions (what you discovered, what is new, and how this advances the field).**

**Response:** We thank the referee for this suggestion.

**Key findings:** The core research findings indicate that consumer services, industrial and basic materials were the most affected as their prices dropped by more than 30 percent during the examined period. In addition, consumer services and industrial and basic materials sectors were identified as highly correlated to the oil price movement. The consumer services sector is directly exposed to oil price movements as government revenues and public spending. Consequently, consumer confidence is significantly affected by lower oil prices, which may soften the demand for consumer services. The industrial sector is considered the most exposed sector to oil prices due to petrochemicals and refining activities that were negatively affected, as lower oil prices translate into less profits. Finally, in the case of basic materials, the cost and availability of raw materials like petrochemicals, fertilisers, and construction materials are also significantly influenced by oil prices, as many of these materials are oil derivatives (IMF, 2022, World Bank, 2023, OPEC, 2023).

**Novelty, and Contributions:** The novelty of this paper is threefold:

a) The study adds to the extant literature through the comprehensive analysis of the Kuwaiti stock market sectors to shed light on their performance through the combined shocks of the COVID-19 pandemic and the oil price war between Saudi Arabia and Russia. To the best of the authors’ knowledge, this dual shock remains underexplored in the academic literature, with a significant lack of studies on the context of Kuwait’s stock market at a sectoral level.
b) The study integrates a solid methodological framework based on traditional and well-established econometric models that enable cross-checking the research findings and provide evidence of their value to understanding sectoral sensitivity to higher levels of uncertainty.
c) The analysis helps to outline critical insights and recommendations for oil-dependent economies within the context of enhanced uncertainty and the imperative of economic diversification within the GCC and the Middle Eastern region.

- **I request the authors to ensure these elements are explicitly and clearly presented in both the abstract and introduction.**

**Response:**
We thank the referees for all their suggestions and comments on how to improve this research paper. We confirm that all comments have been addressed, and changes have been integrated into the research paper. A detailed explanation of required changes is also presented in this document.

The abstract has been revised summarizing the research question, methodology (Johansen Cointegration, Granger Causality, and Breitung-Candelon Frequency Domain models), key findings (30% sectoral drops, long-run WTI relationship, short-run dynamics with Brent, OPEC, Dubai), and novelty (first multi-methodological sectoral analysis in Kuwait). As follows:

This study examines Kuwait’s stock market sectors’ response to the 2020 dual shock of the COVID-19 pandemic and the oil price war between Russia and Saudi Arabia, using traditional cointegration and causality models alongside frequency domain analysis to assess long and short-run dynamics. Daily data from December 31, 2015, to February 23, 2022, reveal that consumer services, industrial, and basic materials sectors dropped by approximately 30 percent during the dual shock, reflecting Kuwait’s oil-dependent economy. The findings highlight a significant long-run relationship with the West Texas Intermediate index (WTI) and short-run dynamics with the Brent crude oil index, the OPEC, and Dubai benchmarks, underscoring the need for the country’s economic diversification. The research findings are relevant to oil-dependent economies as sectoral exposures can increase regional financial instability. At the global level, monitoring the impact of shocks in commodity-driven economies can help in designing policies that minimise the effects of systemic risks, affecting energy supply chains and inflation stability. This research advances the field by providing a multi-methodological analysis integrating traditional and efficient econometric models to assess sectoral sensitivities in the context of Kuwait, which is an understudied oil-exporting economy. The study offers valuable insights for investors and policy-makers in managing risks associated with oil price fluctuations during times of enhanced uncertainty.

The introduction (Section 1) has been restructured and tightened by 50%, reduced from 2,202 words to 1084 words, and is now organized into five clear paragraphs:

- Paragraph 1: Context and significance (2020 dual shock, Kuwait’s oil dependency).
- Paragraph 2: Research gap, question (“What is the impact of the 2020 dual shock on Kuwait stock market sectors?”), and hypotheses (H1A, H1B, H2A, H2B).
- Paragraph 3: Methodology overview (traditional time series models,) and Key findings.
- Paragraph 4: The significance implication of the study, why it’s important considering Kuwait, the Middle Eastern economy, and potentially the global economy.
- Paragraph 5: Novelty, and Contributions (first sectoral study in Kuwait during the dual shock) and paper outline.
  This addresses the referee’s call for clarity and comprehensive coverage of key elements.

As follows:

**Introduction**

The year 2020 was a challenging year for the world’s socioeconomic system due to a dual shock that was defined by a deadly pandemic and oil price instability as a result of the war between Russia and the Kingdom of Saudi Arabia. Oil exporting economies were significantly affected as the economic system entered a period of hibernation forced by health measure restrictions that introduced major constraints through social distancing aiming to limit human interactions. Among these exporting economies is Kuwait, which registered a significant drop in its All-share index by 25 percent on March 23, 2020 (Al-otaibi and Morales, 2022). According to the OPEC Annual Report (2021), Kuwait ranks among the world’s leading oil-exporting nations. In 2020, Kuwait was the world’s eighth largest producer of crude oil, valued at approximately 2,438 million dollars per day, accounting for approximately 4 percent of the world volume of crude oil exports. Moreover, Kuwait accounts for 11 percent of the OPEC member’s value of petroleum export countries, representing approximately 35,231 million US dollars, with 6 percent of the world’s oil reserves, by 101.5 billion barrels. The oil sector makes up nearly half of Kuwait’s GDP, around 95 percent of its exports, and approximately 91 percent of government revenue (OPEC, 2023). The country’s industrial sector accounts for 45.4 percent of its GDP and employs 25 percent of the workforce. In contrast, the manufacturing sector remains underdeveloped, contributing only 7 percent to the national GDP, as reported by the World Bank (2022, 2023). The Kuwait economic model is significantly exposed to the oil market dynamics (Al-Shami and Ibrahim, 2013; Al-Hayky and Naim, 2016; Merza and Almusawi, 2016; Kisswani and Elian, 2017; Kisswani and Elian, 2018; Alshihab and Alshammary, 2020). Researchers such as Al-Yahyaei et al. (2021) and Hammami (2020) have offered evidence that Kuwait’s heavy dependence on oil-made sectors that are tied to oil and gas, such as basic materials and industrials, which are sectors that suffered significant losses resulting from the oil price collapse and the significant economic disruptions resulting from the 2020 Global Health Crisis. During this period, the oil barrels were sold at historically low prices, as WTI recorded a historic low, entering negative territory with a -36$ price that was followed the next day with -6$ for the first time in oil history (Estrada, 2020; IMF, 2022, World Bank, 2023, OPEC, 2023). The importance of oil for Kuwait’s economy is unquestionable, with a need to have insights on implications for its sectors and their stability during times of high uncertainty, as captured in the study’s leading research question and two research hypotheses as follows,

- What is the impact of the 2020 Dual market shock (COVID-19 and oil prices shock) on Kuwait stock market sectors?

Hypotheses:

3. H10: There is evidence of a potential long-run relationship between oil benchmarks and Kuwait’s stock market sectors.

H1A: There is no evidence of a potential long-run relationship between oil benchmarks and Kuwait’s stock market sectors.

4. H20: There is evidence of a potential short-run relationship between oil benchmarks and Kuwait’s stock market sectors.

H1A: There is no evidence of a potential short-run relationship between oil benchmarks and Kuwait’s stock market sectors.

The research methodology integrates well-known and established econometric models such as the Johansen and Juselius Cointegration test (1988), the Engle and Granger Causality test (1987), the Granger causality test (1969), and the Breitung and Candelon Frequency Domain Causality test (2006) models to ensure a robust framework through the implementation of different econometric models aiming to ensure consistency on results. The core research findings indicate that consumer services, industrial and basic materials were the most affected as their prices dropped by more than 30 percent during the examined period. In addition, consumer services and industrial and basic materials sectors were identified as highly correlated to the oil price movement. The consumer services sector is directly exposed to oil price movements as government revenues and public spending. Consequently, consumer confidence is significantly affected by lower oil prices, which may soften the demand for consumer services. The industrial sector is considered the most exposed sector to oil prices due to petrochemicals and refining activities that were negatively affected, as lower oil prices translate into less profits. Finally, in the case of basic materials, the cost and availability of raw materials like petrochemicals, fertilisers, and construction materials are also significantly influenced by oil prices, as many of these materials are oil derivatives (IMF, 2022, World Bank, 2023, OPEC, 2023).

This paper has significant implications for oil-dependent economies. The dual shock of the COVID-19 pandemic and oil price war exposed critical vulnerabilities in mono-economies, revealing the urgent need for economic diversification and sectoral resilience (OPEC, 2023; World Bank, 2022). Kuwait’s experience offers insights into Middle Eastern economies and the GCC countries facing similar oil dependencies (Alshihab & Alshammari, 2020). In particular, the study offers insights into how global shocks are transmitted through financial systems in oil-exporting economies, thus affecting international commodity markets, investment flows, and regional economic stability (Jawadi & Sellami, 2021; Gharib, Mefteh-Wali, & Jabeur, 2020). Understanding sectoral sensitivities improves the ability of policy-makers and investors to manage risks and design adaptive strategies in the face of future global crises (Fernandez, 2020; Algahtani, 2019). As oil price volatility continues to shape global economic performance, especially in emerging markets, our findings contribute to the literature on macro-financial linkages and policy responsiveness in interconnected economies.

The novelty of this paper is threefold:

d) The study adds to the extant literature through the comprehensive analysis of the Kuwaiti stock market sectors to shed light on their performance through the combined shocks of the COVID-19 pandemic and the oil price war between Saudi Arabia and Russia. To the best of the authors’ knowledge, this dual shock remains underexplored in the academic literature, with a significant lack of studies on the context of Kuwait’s stock market at a sectoral level.
e) The study integrates a solid methodological framework based on traditional and well-established econometric models that enable cross-checking the research findings and provide evidence of their value to understanding sectoral sensitivity to higher levels of uncertainty.
f) The analysis helps to outline critical insights and recommendations for oil-dependent economies within the context of enhanced uncertainty and the imperative of economic diversification within the GCC and the Middle Eastern region.

The remainder of the paper proceeds as follows. Section 2 presents the relevant literature; section 3 defines the methodological research framework. The paper’s main results and discussion are presented in sections 4 and 5, and finally, section 6 concludes the paper.

2. **Sections 1.1–1.3 and Section 2 could be easily combined into just one or two pages without losing essential content.**

**Response**:
We have consolidated Sections 1.1–1.3 into a single section within the Introduction (Section 1,). This revision summarises the 2020 dual shock’s global impact, Kuwait’s oil dependency (95% exports, 91% government revenue), and the literature gap on GCC sectoral analysis and hypothesis, Methodology overview, justification and novelty as requested.

3. **Also, the current subdivision of the introduction section does not follow standard academic conventions. I recommend rewriting this section to better align with the coherent structure I outlined in my previous comments (point 1).**

**Response**:
The introduction has been restructured to align with academic conventions (Section 1, 1084 words). It now follows a standard format:

- **Context and Significance**: Introduces the 2020 dual shock and Kuwait’s economic vulnerabilities.
- **Research Gap and Question/Hypotheses**: Clearly state the research questions and hypotheses (H10, H1A, H20, H2A).
- **Methodology Overview**: Justifies the use of Johansen Cointegration, Granger Causality, and Frequency Domain models, noting their suitability for the dataset.
- **The significance of the study**: Clearly states the significant implications of the study, why it’s important considering Kuwait, the Middle Eastern economy, and potentially the global economy.
- **Novelty and contributions and Outline**: Highlights novelty and contribution of the study (first sectoral study in Kuwait) and outlines the paper’s structure. This revision eliminates the previous subdivided structure (Sections 1.1–1.3) and ensures a cohesive, conventional flow.

4. **Section 3 contains explanations and contents that could be moved to an appendix (e.g., methodological details) since it does not add any new insight to the readers and to the core narrative. Section 3.1.3 (Descriptive Analysis) should instead follow immediately after the data description for better flow.**

**Response**:
We have reorganised Section 3 (now Section 3 in the revised manuscript, 1,500 words) to improve flow and clarity:

- **Methodological Details**: Detailed descriptions of Stationarity (Section 3.2.1), Cointegration (Section 3.2.2), Causality (Section 3.2.3), and domain Causality (Section 3.2.4) have been moved to Appendix 2 (Methodological Framework, around 1,663 words). Section 2.3 now provides a brief overview of these methods, linking them to hypotheses.
- **Descriptive Analysis Placement**: Section 3.1.3 (Descriptive Analysis) has been integrated into Section 3.2, immediately following Section 3.1 (Data Description). This section now includes summaries of Tables 2 and 3, focusing on key insights (e.g., healthcare’s highest mean return, WTI’s highest volatility) without redundant notes.
- **Data Description**: Section 3.1 focuses on data sources (DataStream, 2015–2022) and sector/benchmark details (Table 1). This reorganisation enhances logical flow and adheres to the referee’s suggestion.

5. **Tables 2 and 3 do not appear to provide significant insights beyond what is already stated in the text. The accompanying notes under each Tables merely repeat mechanical explanations found in the main body, making them redundant.**

**Response**:
Tables 2 and 3 have been retained in the main text (Section 3.2) but revised to address redundancy:

- **Simplified Notes**: Table notes are now concise: “Summary statistics of daily returns and prices for Kuwait stock market sectors (Table 2) and oil benchmarks (Table 3) from December 31, 2015, to February 23, 2022. Source: DataStream (2022).”
- **Focused Text**: The accompanying text emphasises key insights (e.g., healthcare’s mean return of 0.000529, WTI’s volatility of 3.17%) without reiterating table data.
  These changes ensure that Tables 2 and 3 provide meaningful insights while eliminating repetitive content, as requested.

6. **Please remember, “*The quality of a paper is monotonically decreasing in length –shorter is better, everything else held constant.”* I strongly recommend tightening all sections to focus on key points efficiently.**

**Response**:
The manuscript has been restructured and tightened by approximately 20–25%, reduced from 9,296 words to 7,187 words (excluding appendices, tables, and references that are needed to ensure that readers have all required information supporting the research study). Specific actions include:

- **Introduction restructured and tightened:** The introduction (Section 1) has been restructured and tightened by 50%, reduced from 2,202 words to 1084 words.
- **Streamlined Sections**: The Introduction (Section 1) and Data and Methodology (Section 3) are more concise, with methodological details moved to Appendix 2.
- **Focused Discussion**: Sections 4 (Results) and 5 (Discussion) emphasise key findings and contrasts without excessive elaboration.
  This revision maintains quality and depth while adhering to the referee’s call for brevity.

7. **The section titled “*3.1.1 The Research Question and Hypothesis*” needs to be revised, as the key hypotheses and the final description do not align well with the introduction. Additionally, there is unnecessary repetition of content that was already covered in the introduction.**

**Response**:
Section 3.1.1 has been revised and integrated into Section 2.1 (Data Description) to align with the Introduction (Section 1):

- **Hypotheses Clarity**: Hypotheses (H10, H1A, H20, H2A) are clearly listed and linked directly to the research question, avoiding repetition of background details from the introduction.
- **Alignment**: The revised section ensures consistency with the introduction’s research question and hypotheses, focusing on data relevance to testing long- and short-run relationships.
  This addresses the referee’s concern about misalignment and repetition.

8. **Based on my understanding of the introduction and hypotheses, the sections *3.2.1 Stationarity, 3.2.2 Cointegration, and 3.2.3 Causality* should either be moved to the appendix or revised to better align with the research questions/hypothesis. You need to explain how each of these very basic time series concepts is connected to the hypotheses and whether they support or refute them.**

**Response**:
Sections 3.2.1–3.2.4 (Stationarity, Cointegration, Causality) have been moved to Appendix 2 (Methodological Framework, words) to reduce main text length while maintaining technical rigor. A brief summary in Section 2.3 (Methodology Overview) now connects these methods to the hypotheses:

- “Stationarity tests (ADF, PP, KPSS) confirmed series stationarity in returns, supporting H20/H2A. Cointegration (Johansen) and causality (Granger) analyses tested H10/H1A and H20/H2A, respectively, with frequency domain methods enhancing short-run insights.”
  This revision ensures clear alignment with hypotheses while addressing the referee’s suggestion to streamline the main text.

9. **I noticed that there is a contrast between your findings and the previous studies discussed in *section 5, “Critical Discussions*.” It is important to explain why your findings differ from previous research and highlight any new insights that support the conclusions of earlier studies.**

**Response**:
Section 5 has been updated to explicitly contrast findings with prior studies and highlight new insights:

- **Contrasts**: In contrast, the research study developed by Kisswani and Elian (2017) illustrates a long-run relationship between Kuwait stock market returns and both oil prices (Brent and WTI) in which the daily oil price shocks have a negative impact on stock returns. This research study illustrates that the Kuwait stock market is cointegrated with only WTI, and there is no evidence of cointegration with the Brent oil benchmark. In addition, the causality test shows that financial services, basic materials, and real estate are causal, along with all oil benchmarks. It also aligns with Alshihab and Alshammari (2020) on oil-stock dynamics but extends their work with sectoral granularity.
- **New Insights**: Basic Materials’ basic materials sector has long-run and short-run dynamics with all oil benchmarks. This illustrates that the behaviour of oil benchmark price highly influences the basic materials sector, while insurance and technology’s lack of causality suggests resilience, offering novel implications for risk management.

The novelty and contributions of this paper are fulfilling three research gaps:

- There is a dearth of research studies that understand the relationship between crude oil prices and the stock market during the dual market shock. COVID-19 and oil price shocks provide valuable advantages for investors and policy-makers regarding both markets’ behaviour. It provides an important tool during times of significant distress, such as the dual market shock (COVID-19) and oil price shock, mainly through investment and hedging activities as knowledge gained from sectoral sensitivity to the shock.
- There is a lack of research studies that combine multiple methodologies such as the Johansen and Juselius Cointegration test (1988), Engle and Granger Causality test (1987), Granger causality test (1969), and Breitung and Candelon Frequency Domain Causality test (2006) focused on Kuwait’s stock market at a sector level. By implementing a multiple methodologies approach, this research provides insights for investors, portfolio managers, policy-makers and academics interested in the analysis of sectoral behaviour and sensitivity of the Kuwaiti stock market in relation to oil benchmarks (Brent, Dubai, OPEC, and WTI). This study can assist investors in diversifying their portfolios and recognising the importance of hedging against market uncertainties. The combination of multiple methodologies enhances understanding of the dual shock’s effects on Kuwait’s stock market. Such insights are valuable for investors targeting oil-dependent economies like Kuwait, allowing for a multidimensional view and offering a comprehensive insight into the impact of a dual shock in Kuwait’s stock market. The econometric working framework enhanced the analysis while highlighting the comparative strengths of both well-established econometric models.
- There is a dearth of research studies examining the dynamic relationship between oil prices and Kuwaiti Stock Market sectors amidst the dual shock. This paper adds value to the literature through the comprehensive analysis of Kuwaiti stock market sectors to shed light on how they responded to the combined shocks of the COVID-19 pandemic and the oil price war between Saudi Arabia and Russia. To the best of the authors’ knowledge, this dual shock remains underexplored in the academic literature, with a significant lack of studies on the context of Kuwait’s stock market at a sectoral level.

These additions address the referee’s request for clearer differentiation and contributions.

10. **The revised *manuscript with track changes* does not adhere to the standard manuscript submission style. Please ensure that this is addressed in future submissions.**

The research paper has been updated based on the feedback received. The manuscript without track changes has been submitted to ensure that the given comment is addressed.
