# Supplementary material for: Sectoral sensitivity of the Kuwait stock market to a dual shock
Source: PLoS One. 2025 Sep 24;20(9):e0331384. doi: 10.1371/journal.pone.0331384 (PMC12459840; doi:10.1371/journal.pone.0331384)
Supplement: Attachment — Submitted filename: Reviewer Report -PONE-D-23-38848.docx [file pone.0331384.s021.docx]

This manuscript presents a comprehensive and timely examination of Kuwait's stock market sectoral response to the dual shock of the COVID-19 pandemic and the 2020 oil price war. The study tends to be original and methodologically sound. It will also be highly relevant to academic researchers and policy-makers interested in the dynamics of oil-dependent economies. The paper addresses a clear gap in the literature, providing a sectoral-level analysis of an underexplored context (Kuwait) about a significant dual economic health crisis. Using Johansen Cointegration, Granger Causality, and Frequency Domain Causality provides a robust multi-angle approach. Strong empirical analysis highlighting differential sector responses (e.g., Basic Materials, Industrials, Consumer Services) led to the effective use of figures and tables to visualize market drops. Also, the findings will be helpful for investment strategy and policy design, especially regarding risk management and economic diversification in oil-dependent economies. However, some clarity still needs to be sorted out.

### **Revisions Required**

### (1). Redundancy: Reduce repetition across Introduction, Discussion, and Abstract (e.g., repeated reporting of sectoral drops). The formatting needs to be improved, especially for sector drop figures, to ensure consistency with axis scales, labelling, and legends. Also, the author should summarize key takeaways below descriptive tables to aid interpretability.

(2). Optionally, compare findings with other GCC economies to enhance regional relevance.

(3). Although the rationale for avoiding robustness checks on residuals is justified, a clearer explanation (or relegation to the Appendix) would help clarify.

(4). Address minor typographical issues (e.g., inconsistent spacing, variable formatting) and revise for brevity where sections become overly lengthy.
